# Targeting CDK7 suppresses super enhancer-linked inflammatory genes and alleviates CAR T cell-induced cytokine release syndrome

**DOI:** 10.1186/s12943-020-01301-7

**Published:** 2021-01-04

**Authors:** Ye Wei, Chong Li, Huifang Bian, Wei Qian, Kairui Jin, Tingting Xu, Xiaomao Guo, Xueguan Lu, Fengtao Su

**Affiliations:** 1 Department of Radiation Oncology, Cancer Institute, Fudan University Shanghai Cancer Center, Shanghai, 200032 China; 2 Department of Oncology, Shanghai Medical College, Fudan University, Shanghai, 200032 China; 3 Cancer Institute, Fudan University Shanghai Cancer Center, Shanghai, 200032 China; 4 Department of Radiation Oncology, Fudan University Shanghai Cancer Center, Shanghai, 200032 China

**Keywords:** Cyclin-dependent kinase 7, Super enhancer, Inflammatory genes, Cytokine release syndrome, Macrophages, Chimeric antigen receptor engineered T cells

## Abstract

**Background:**

Cytokine release syndrome (CRS) is a systemic inflammatory response characterized by the overexpression of inflammatory genes. Controlling CRS is essential for improving the therapeutic effects of chimeric antigen receptor (CAR) engineered T cells. However, current treatment options are limited given the complexity of cytokine interactions so it is important to seek a mild strategy with broad-spectrum inhibition to overcome this challenge.

**Methods:**

Using THZ1, a covalent inhibitor of cyclin-dependent kinase 7 (CDK7), we demonstrated the transcriptional suppression of inflammatory genes in activated macrophages. RNA sequencing and ChIP sequencing were conducted to identify the key target genes of the inflammatory response. Pathogen- and CAR T cell-induced CRS models were also established to assess the efficacy and safety of targeting CDK7.

**Results:**

CDK7 blockade attenuated cytokine release, mitigated hyperinflammatory states and rescued mice from lethal CRS. Targeting CDK7 preferentially suppressed a set of inflammatory genes, of which STAT1 and IL1 were the key targets associated with super enhancers. Furthermore, we confirmed the potent efficacy of THZ1 in alleviating the CRS induced by CAR T cell infusion without causing tissue injury or impairing antitumor effects.

**Conclusions:**

Our work indicates the CDK7-dependent transcription addiction of inflammatory genes. Targeting CDK7 is a promising strategy for treating CRS by inhibiting multiple cytokines.

**Supplementary Information:**

The online version contains supplementary material available at 10.1186/s12943-020-01301-7.

## Background

Cytokine release syndrome (CRS) is a life-threatening inflammatory disorder that can be triggered by infections or immunotherapies [1]. Severe CRS is often reported in some cases of acute inflammatory responses and is characterized by fever, hypotension, and respiratory insufficiency. The great progress in immunotherapy in the last decade has, to some extent, contributed to the high incidence of CRS [2, 3], especially the adoptive transfer of chimeric antigen receptor engineered T cells (CAR T), which has emerged as a novel approach for leukemia and lymphoma, with a reported incidence of severe CRS ranging from 20 to 50% in different trials [4–6]. Once the inflammatory disorder initiates, elevated levels of serum cytokines, including interferons (IFNs), interleukins (ILs), chemokines, colony-stimulating factors (CSF) and tumor necrosis factors (TNF), will constantly stimulate immune and stromal cells, gradually enter a proinflammatory loop and develop into multiorgan injury when the reaction loses control to destroy the immunologic homeostasis [1].

Despite the controversy over the immunopathological basis, macrophage overactivation is essential to maintain the hyperinflammatory state in CRS. From innate to adaptive immunity, macrophages respond to stimuli by promoting multi-cytokine release and interacting with different cell types [7]. IL-6 and IL-1β, mainly derived from macrophages, have been regarded as the driving force of CRS and make contributions to related injuries. Especially in CAR T models, evidence has shed light on that the severity of CRS is not totally mediated by cytokines derived from CAR T cells, but by factors derived from the recipient macrophages [8, 9]. This conclusion is supported by several studies focused on CAR T therapy-induced encephalopathy, a delayed neurotoxicity which had previously been ascribed to the cytokine release of CAR T cells [10]. Data from murine models also support the causal association of CAR T-induced neurotoxicity with macrophage-derived IL-1β [9]. Even today, systemic corticosteroids are widely used to attenuate CRS symptoms, and prolonged-use/high-dose corticosteroids may result in immunosuppression, secondary infection, as well as ablation of CAR T cell expansion and persistence [11]. Recently, encouraging results from trials have accelerated the approval of inhibitors targeting IL-6 or IL-1 signaling by virtue of their inhibition of inflammation [12]. However, blocking specific cytokines is also hardly sufficient because of the complex crosstalk within the cytokine storm [13]. Thus, seeking a mild anti-inflammatory strategy with broad-spectrum inhibition is necessary to overcome this challenge.

THZ1 is a covalent inhibitor of cyclin-dependent kinase 7 (CDK7) and suppresses gene transcription by dephosphorylating the C-terminal domain of RNA polymerase II (RNA Pol II) [14]. Differing from the conventional understanding of blocking CDK7, which is expected to adversely affect global transcription, THZ1 preferentially impacts a set of critical genes regulated by super enhancers (SEs). The selectivity of transcriptional repression mediated by THZ1 is linked to CDK7-dependent enhancer remodeling, which is a possible explanation for the transcription addiction of identity genes and has been thoroughly identified in MYCN-amplified neuroblastoma [15], triple-negative breast cancer [16], T cell lymphoma [17] and small cell lung cancer [18]. More interestingly, THZ1 is being taken seriously as an immunoregulator because blocking CDK7 has been shown to manipulate the intension and resolution of inflammation by reinvigorating antitumor immunity [19] and regulating granulocyte apoptosis as well as cytokine secretion [20]. In addition, RNA Pol II-driven transcription can be disrupted with topoisomerase 1 inhibitor to suppress inflammatory genes and protect against lethal CRS [21]. These findings indicate that cell- and gene-specific effects can be observed even when targeting a so called “general transcriptional regulator” and CDK7 appears to be a promising target to achieve selective transcriptional inhibition. Thus, blocking CDK7 may be a feasible strategy to inhibit the overexpression of various inflammatory genes in CRS if we could identify the SEs responsible for the inflammatory processes.

Here, we demonstrate that pretreatment with THZ1 significantly reduces the massive release of multi-cytokines and alleviates hyperinflammatory symptoms. The efficacy and safety of THZ1 are confirmed in bacteria- or virus-induced inflammatory responses and reduplicated successfully in CRS triggered by CAR T infusion without dampening antitumor capability. Mechanistic exploration further reveals a key role of STAT1/IL1 in the inflammatory response, which is vulnerable to THZ1 treatment under the control of SEs. Our work provides a promising strategy with broad-spectrum inhibition for current CRS treatment.

## Materials and methods

### Cells and virus

Peripheral blood mononuclear cells (PBMCs) were separated from the blood samples of 4 healthy volunteers after gradient centrifugation, and cultured in DMEM for adhesion purification. After 2–4 h, suspended lymphocytes were pooled and activated with anti-CD3/CD28 for 1 day and then expanded in DMEM 10% FBS supplemented with 2 U/ml IL-7/IL-15 to obtain proliferative T cells.

Two forms of macrophages were used in our experiments: Adherent fractions of PBMCs were cultured in DMEM 10% FBS supplemented with 20 ng/ml rhGM-CSF for 7 days to induce primary monocyte-derived macrophages (MDMs); PMA-induced macrophages (noted as mTHP-1) were obtained from human monocyte THP-1 cells via a 24-h incubation with 100 ng/ml PMA and cultured in PMA-free medium overnight to reduce their inflammatory baseline.

Burkitt lymphoma Raji cells and human fibroblast HFF-1 cells were respectively cultured in RPMI-1640 and DMEM containing 10% FBS and 50 U/ml penicillin-streptomycin. Human umbilical vein endothelial cells (HUVEC) were cultured in endothelial basal medium supplemented with EGM-2 MV Single-Quots (EGM-2 MV medium, Lonza).

The influenza virus H1N1 (strain A/California/07/2007) was used as the viral stimulus to activate macrophages.

### Quantitative real-time PCR analysis

MDMs derived from 3 healthy individuals or mTHP-1 cells were treated with THZ1 for 0.5–1 h before or after stimulation. Total RNA was extracted from cell lysates and reverse transcribed to cDNA using a reverse-transcriptional kit (TaKaRa). RT-PCR was performed in a triplicate with the SYBR Prime Script RT-PCR kit (TaKaRa) on the 7300 plus Real-Time PCR system (Applied Biosystems). The primer sequences are described in the Supplementary Materials and Methods.

### RNA sequencing and analysis

MDMs derived from 1 healthy individual were divided into three parts: the control cultured with medium, the stimulated cells cultured with LPS, and the rescued cells pretreated with 30 nM THZ1 for 0.5 h prior to LPS stimulation. Total RNA was prepared as described in the quantitative real-time PCR experiments. The isolated RNA was used to prepare RNA-seq libraries using TruSeq RNA Library Prep Kit v2 (Illumina) following the manufacturer’s instruction. The libraries were sequenced on an Illumina HiSeq X-ten sequencing System with a read length of 150 base pairs.

Raw RNA-seq reads were first preprocessed the in-house Perl scripts and sickle software (version, 1.200). The preprocessed reads were aligned against the human genome (GRCH38, https://asia.ensembl.org/info/data/ftp/index.html) using HISAT2 (v2.1.0) [22]. Gene-level read counts were summarized with HTSeq script (version 0.6.0) [23]. Genes with more than 50 reads in at least one library were retained and used to identify DEGs with the Bioconductor package DEGseq [24]. A DEG met the following criteria: average expression abundance of the gene at least in one sample more than 1 Fragments per Kilobase Million (FPKM), false discovery rate (FDR, an adjusted *P* value after multiple testing of Benjamini-Hochberg [25]) < 0.01 and abs (fold change) > 2. Heatmaps in Fig. 3a were plotted using heatmap.2 in gplots, and Fig. 3d was created using the Heatmap script in R package ComplexHeatmap.

The DAVID suite of online tools (http://david.abcc.ncifcrf.gov/tools.jsp) was used to determine the biological process ontology defined by the Gene Ontology Consortium. Gene Set Enrichment Analysis was performed using a gene list pre-ranked by fold change upon LPS treatment from mTHP-1 cells. The network in Fig. 3e was created using the functional Network in R package FGNet.

### ChIP sequencing and analysis

PMA-induced mTHP-1 cells were treated as described in RNA-seq. Samples for ChIP-seq were prepared according to the manufacture’s instructions (SimpleChIP Enzymatic Chromatin IP Kit, CST). ChIP-seq libraries were constructed using NEBNext Ultra II DNA Library Prep Kit for Illumina (NEB). We used the MACS2 (v2.2.6) peak finding algorithm to identify regions of ChIP-seq enrichment over background for all ChIP-seq data sets with a *P* value of 1e-9. For H3K27ac data, we pooled all samples and their input DNA to identify consensus enriched region. The consensus enriched regions were used as constituent enhancers for ROSE to identify super enhancers and separate them from typical enhancers. The software was run with parameters of -s 12500, −t 2000, and -g hg38 to rank enhancers by the H3K27ac signal minus the input DNA control signal. Stitched enhancers were assigned to the associated genes based on geneMapper in ROSE and read density calculations were performed using bamToGFF with parameters -e 200, −m 1, −r, and -f 1 [26]. Read density was defined as units of reads per million mapped reads per base pair (rpm/bp). We used deeptools to plot ChIP-seq heatmaps in Fig. 3g and Fig. S3B. Analysis of motif enrichment was performed using AME (http://bioinformatics.org.au/ame/). All sequencing data are available in the SRA database with BioProject (PRJNA635530).

### Generation of CAR T

CARs are composed of an extracellular single-chain variable fragment (scFv), which serves as the targeting moiety, a transmembrane spacer, and an intracellular signaling/activation domain(s). The 2nd-generation CARs also contain costimulatory domains such as CD28 and 4-1BB. Lentiviral vectors of CD19 scFv-4-1BB-CD3ζ were designed and used in our experiments. Primary T cells were infected with lentivirus (MOI=10) after activation with anti-CD3/CD28, and expanded in the presence of 2 U/ml IL-7/IL-15 for 2 weeks. Infection efficiency and hallmarks of T cells were detected by flow cytometry. T cells without lentiviral infection were defined as the control (negative control T cells, NCT). Both CAR T cells and NCT cells were derived from the same volunteer to minimize the effects of individual differences.

### CAR T functional assays

CAR T cells were cocultured with Raji target cells at the indicated E/T ratio in DMEM 10% FBS for 1 day. The elimination index of Raji cells was calculated using flow cytometry as follows: 1- (number of residual target Raji in the presence of CAR T cells)/ (number of residual Raji in the presence of NCT cells). Coculture supernatants were collected to assess the cytokine release or used as the conditioned medium for macrophages. Subsequently, the conditioned medium was collected after a 6-h or a 24-h incubation with macrophages for further gene expression or cytokine assessment, respectively. In the CFSE assay, CAR T or NCT cells were stained with CFSE in advance and stimulated with Raji at an 8:1 E/T ratio for 3 days, and the proliferation of T cells was measured via flow cytometry.

### Mouse treatment

In the model of LPS-induced CRS, 6-week-old female C57BL/6 mice were intraperitoneally injected with a dose of 10 mg/kg of THZ1 at 0.5 h before LPS treatment. In the model of CAR T-induced CRS, 6-week-old female SCID-Beige mice were intraperitoneally injected with 3× 10^6^ Raji cells for 3 weeks before intraperitoneal transfer of 3× 10^7^ CAR T cells [9]. THZ1 was administered 3 h prior to CAR T or NCT infusion, and this administration was repeated twice at 12, and 24 h after infusion. To assess CRS toxicities, mice were monitored for daily activity, anal temperature, weight loss and mortality. Blood samples were collected by retro-orbital bleeding and left to clot for 30 min at room temperature. Serum was isolated via centrifugation and stored at − 80 °C. To detect the level of intraperitoneal myeloid cells, PBS was injected into the peritoneum to extract the lavage from mice for flow cytometry. In LPS models, peritoneal macrophages were sorted to detect the transcription of inflammatory genes. Lung, liver, kidney, spleen and dorsal skin were removed, formalin-fixed and paraffin-embedded for histological analysis. All experiments in vivo were approved by the Ethical Committee of Fudan University Shanghai Cancer Center and were compliant with all relevant standards.

### Flow cytometry

For the analysis of cytokines, serum in vivo and supernatant in vitro were diluted in the proper proportion and assessed with the Mouse or Human Anti-Virus Response Panel (Biolegend). For the analysis of peritoneal lavage, samples were processed with erythrocyte lysis buffer and then gently dissociated through a 70-μm strainer to prepare single cell suspensions. Dead cells were excluded with zombie dye and suspensions were labeled with anti-CD86, anti-CD11b, anti-F4/80, anti-Ly6C, anti-Ly6G. In the CAR T generation experiments, T cells infected with lentivirus were stained with anti-CD25 and anti-CD69 to evaluate the activation of T cells at day 2, and with anti-CD4 and anti-CD8 to evaluate the distribution of subsets weekly. For the analysis of the elimination index of Raji cells, cocultured cells were stained with anti-CD3, anti-CD19 and zombie dye to distinguish T cells and residual living Raji cells. In the apoptosis analysis, cells were tested using FITC-Annexin V/PI kits or 7AAD-Annexin V/PI kits. All the above kits and antibodies were purchased from Biolegend and the labeling performed according to the manufacturer’s protocols.

### Statistical analysis

Statistical analyses were performed using Prism 8. The two-tailed Student’s t test, one-way or two-way ANOVA were performed for experiments with two groups or more than two groups, respectively. The log-rank Mantel-Cox test was performed to assess the statistical significance of Kaplan-Meier survival curves. Detailed information is provided in the figure legends. *P* < 0.05 was considered a statistically significant difference.

## Results

### CDK7 regulates inflammatory genes in activated macrophages

**Fig. 1:**
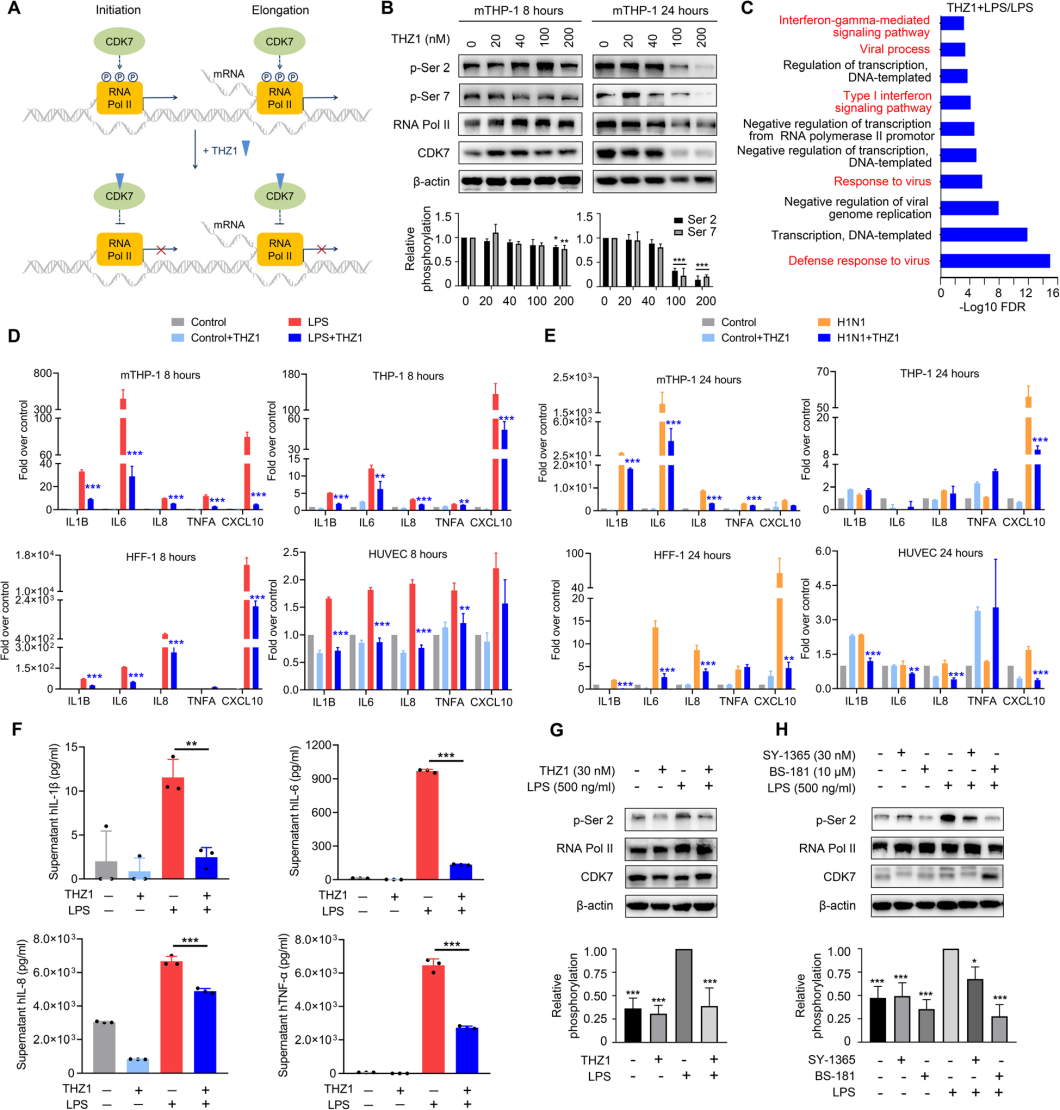
Blocking CDK7 suppresses the inflammatory response to stimuli. **a** Schematic of transcriptional initiation and elongation. CDK7 regulates the transcriptional process by phosphorylating RNA Pol II. **b** Western blot analysis of RNA Pol II phosphorylation and CDK7 expression in mTHP-1 cells treated with the indicated concentrations of THZ1 (0, 20, 40, 100, 200 nM) for the indicated times. **c** Top 10 enriched GO biological processes based on RNA-seq from LPS-stimulated MDMs pretreated with or without 30 nM THZ1. The bar diagram displayed the enriched GO biological processes in THZ1-pretreated cells versus LPS-stimulated cells. **d** Transcriptional levels of inflammatory genes in response to LPS in cells pretreated with 30 nM THZ1 at 8 h after stimulation. **e** Transcriptional levels of inflammatory genes in response to H1N1 infection in cells pretreated with 30 nM THZ1 at 24 h after stimulation. **f** MDMs were stimulated with LPS in the presence of 30 nM THZ1 for 8 h, and supernatant was collected to test the release of cytokines. **g** Western blot analysis of RNA Pol II phosphorylation and CDK7 expression in mTHP-1 cells pretreated with 30 nM THZ1 at 6 h after stimulation. **h** Western blot analysis of RNA Pol II phosphorylation and CDK7 expression in mTHP-1 cells pretreated with 30 nM SY-1365 or 10 μM BS-181 at 6 h after stimulation. Data are the mean ± SD, *n* = 3 or 4 in **b**, **d** to **h**. ***P < 0.001, **P < 0.01, and *P < 0.05 by one-way ANOVA in **d** to **h** or two-way ANOVA in **b**

CDK7 phosphorylates the RNA Pol II in a dynamic, tightly regulated manner to control the initiation and elongation of gene transcription (Fig. 1a). To verify the impact of blocking CDK7 on inflammation, PMA-induced macrophages (mTHP-1 cells) were first treated with a concentration gradient of THZ1 to determine its safe range. At concentrations greater than 100 nM, global transcription was repressed with a considerable decrease in substrate-level phosphorylation of initiation-associated serine 7 and elongation-associated serine 2 (Fig. 1b). Substantial apoptosis was also observed after exposure to concentrations above the threshold and appeared to be concomitant with the decrease in RNA Pol II phosphorylation (Fig. S1A, B). In contrast, very little impact was observed on cells treated with a low dose of THZ1 (≤ 40 nM), supporting the safe range of THZ1 without global transcription shutdown. To avoid the impact of cytotoxicity at high concentrations of THZ1, all subsequent in vitro experiments were performed at concentrations (20–30 nM) suitable for the corresponding cells.

Next, MDMs were treated with 30 nM THZ1 to detect the gene profiles after overexposure to LPS for 8 h. In addition to transcriptional repression, Gene Ontology (GO) enrichment analysis of differentially expressed genes (DEGs) revealed a wide inflammatory inhibition compared with cells that were exposed to LPS (Fig. 1c, Supplementary Table). The expression fluctuation of representative genes, such as IL1B, IL6, IL8 and TNFA, was validated in mTHP-1 and MDMs (Fig. 1d-f, Fig. S1C). Given the wide communications among different cell types and their exposure to massive cytokines [27], THP-1 monocytes, HFF-1 fibroblasts and HUVEC endothelial cells were used to assess the protective effects of THZ1. LPS or influenza virus H1N1, both of which are known as strong inducers of CRS, was used to evoke immune defense against bacterial or viral stimuli, respectively. As expected, a striking amplification of inflammatory genes was observed in immune cells after exposure to LPS/H1N1, in addition to a moderate upregulation in stromal cells (Fig. 1d, e), among which macrophages were the vulnerable cell type after stimulation. Despite the differences in transcriptional reactions, THZ1 universally suppressed inflammatory genes across different cells and presented the strongest protection in macrophages. This difference in susceptibility indicated a cellular selectivity of THZ1 albeit all cell types could more or less benefit from THZ1 treatment. Additionally, some research supports the conclusion that preincubation of THZ1 increases the inhibitory activity of CDK7 and results in a better therapeutic effect of preventive administration [28]. To evaluate the effects of different administration routes, we further compared the inflammatory reaction of macrophages in preventive and therapeutic models. Whether pre- or post- treatment, THZ1 markedly repressed the transcription of inflammatory genes in the early stage of the inflammatory response, but preemptive use of THZ1 exerted stronger protection in response to the higher dose of LPS and this effect became more persistent over time (Fig. S1C, D). Preemptive treatment of THZ1 seemed to help blunt the transcriptional activity of vulnerable genes in the vulnerable cells. Reduction of cytokine release with THZ1 pretreatment further reinforced the remission of inflammation (Fig. 1f). To confirm the involvement of CDK7 in the inflammatory response, we double-checked the effect of blocking CDK7 in activated macrophages with two other highly selective CDK7 inhibitors, SY-1365 and BS-181. Consistent with the observations in THZ1, both SY-1365 and BS-181 significantly suppressed the inflammatory response of macrophages at concentrations that did not cause transcriptional shutdown or cellular damage (Fig. S1A, B, E). The detection of protein levels revealed that a low dose of CDK7 inhibitors was sufficient to prevent the excessive RNA Pol II phosphorylation after stimulation (Fig. 1g, h), which restricted the expression of inflammatory genes and blunted the reaction of macrophages. All these data highlight the potential of targeting CDK7 to attenuate the overwhelming inflammatory response.

### Targeting CDK7 protects against the inflammatory response

**Fig. 2:**
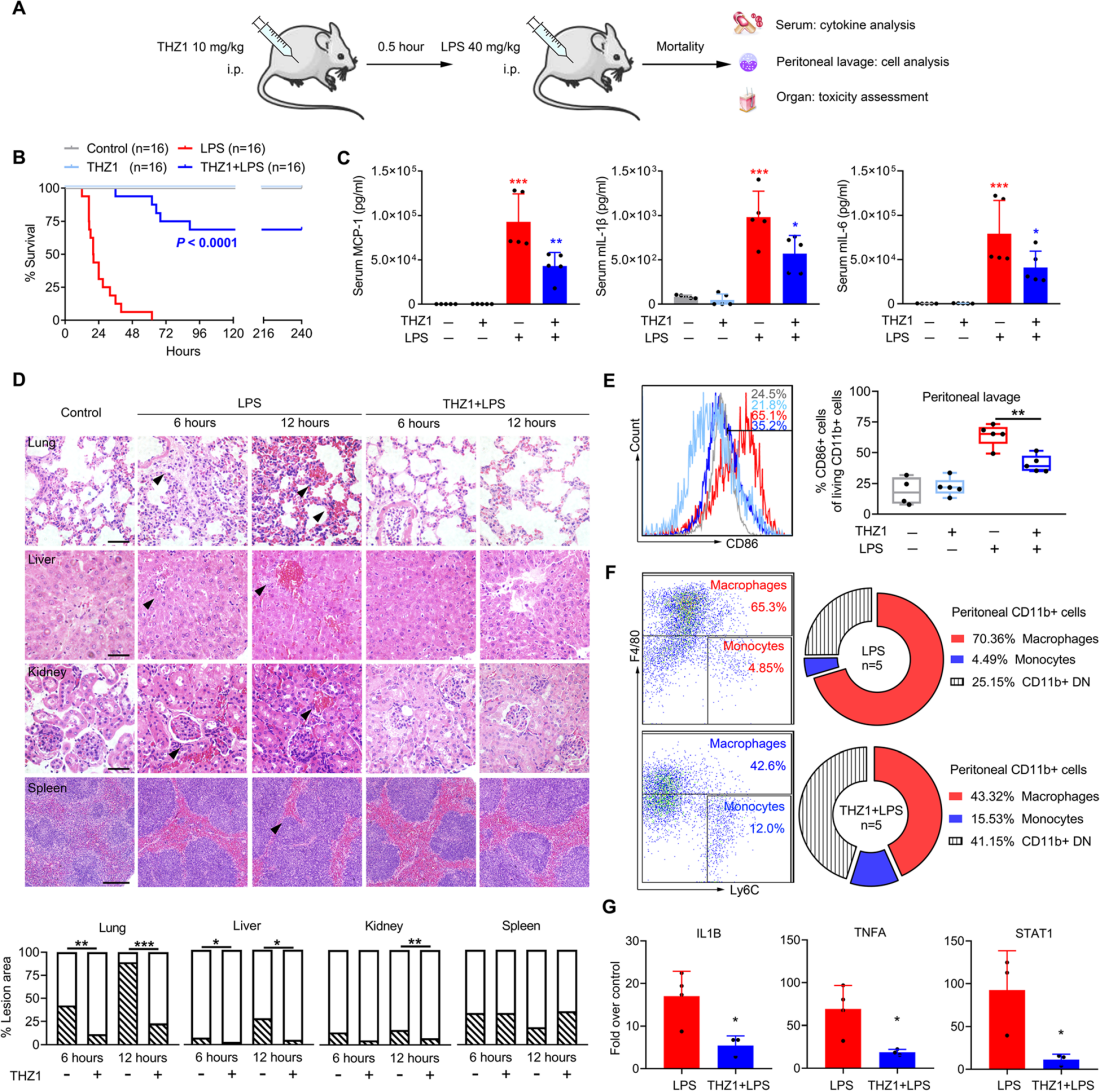
Targeting CDK7 protects against the LPS-induced inflammatory response. **a** Schematic of the murine CRS model triggered by LPS. LPS was intraperitoneally injected following THZ1 pretreatment. **b** Survival of mice in 3 independent experiments (*n* = 16). A log-rank Mantel-Cox was performed for statistical analysis. **c** Serum levels of murine cytokines from mice at 12 h following LPS stimulation. **d** Tissue sections were obtained from mice after THZ1 pretreatment and LPS stimulation and stained with H&E. **e** Representative plots and percentages of living CD11b+CD86+ myeloid cells in peritoneal lavage. **f** Representative plots and percentages of living F4/80+ macrophages, Ly6C+ monocytes and F4/80-Ly6C- (double negative, DN) myeloid cells in peritoneal lavage. **g** Transcriptional levels of IL1B, TNFA, STAT1 in sorted peritoneal macrophages from mice pretreated with THZ1 at 12 h following LPS stimulation. Data are the mean ± SD, n = 3 or 5 in **c** to **g**. ***P < 0.001, **P < 0.01, and *P < 0.05 by one-way ANOVA in **c** and **e** and unpaired *t* test in **d** and **g**

The protective effects of THZ1 inspired us to extrapolate its therapeutic potential in vivo. Therefore, we developed a murine CRS model with LPS to evaluate whether preventive injection of THZ1 could rescue mice from endotoxic shock (Fig. 2a). To optimize the lethal CRS establishment, LPS was injected in C57BL/6 mice with a dose gradient of LPS. Typical symptoms of endotoxic shock occurred at a dose up to 40 mg/kg and led to rapid mortality (Fig. S2A), so we chose this dose to induce lethal CRS in the following experiments. To ensure the THZ1 dosage with minimum toxicity, we used THZ1 at the dose of 10 mg/kg, which had been widely adopted with good tolerance in terms of body weight and blood counts after continuous administration [16, 17]. Previous pharmacokinetic research had revealed a short half-life of THZ1 in plasma samples, with a T1/2 of approximately 45 min [16]. Combined with the prolonged inhibition of covalent binding, this feature might provide a large advantage of THZ1 pretreatment in controlling CRS: short exposure but long-acting. Here, we assessed the therapeutic effect with just a single injection of THZ1 that was much lower than the total dose used in previous reports. As expected, CRS signs including shiver, malaise, and piloerection, appeared rapidly in mice receiving LPS, leading to eventual mortality within 72 h. By contrast, pretreatment efficiently mitigated the shock symptoms and rescued 68.75% (11/16) of the mice after LPS injection (Fig. 2b) with no obvious toxicity in mice treated with THZ1 (Fig. S2B). The increased serum levels of murine IL-1β (mIL-1β), mIL-6 and MCP-1 which correlated well with the severity and survival of CRS, could be significantly attenuated by THZ1 (Fig. 2c). The protection provided by inhibiting CDK7 was also supported by histopathological examinations because inflammatory exudation and hemorrhage were alleviated in multiple organs at 6 and 12 h after THZ1 treatment (Fig. 2d).

To evaluate the immune activation at the cellular level, peritoneal lavage was collected to detect CD86, a marker widely expressed in activated myeloid cells. The brisk elevation of peritoneal CD11b+CD86+ cells, which was already distinct 12 h after LPS injection, was almost halved in mice pretreated with THZ1 (Fig. 2e, Fig. S2C). This phenomenon was further supported by the distribution and response of peritoneal macrophages, which changed markedly in pretreated mice with the decreased percentage of CD11b+F4/80+ macrophages (mean ± SD, LPS, 70.36 ± 6.284; THZ1+LPS, 43.32 ± 11.96) and inhibited transcription of inflammatory genes (Fig. 2f, g, Fig. S2C). A slight increase in CD11b+Ly6C+ monocytes (mean ± SD, LPS, 4.494 ± 3.192; THZ1+LPS, 15.53 ± 10.10) might be a secondary consequence of reduced macrophages. Collectively, the decrease and dysfunction of macrophages support the feasibility for controlling the magnitude of CRS and rescuing mice by blocking CDK7.

### CDK7 inhibitor suppresses the gene profiles of inflammation via RNA pol II

**Fig. 3:**
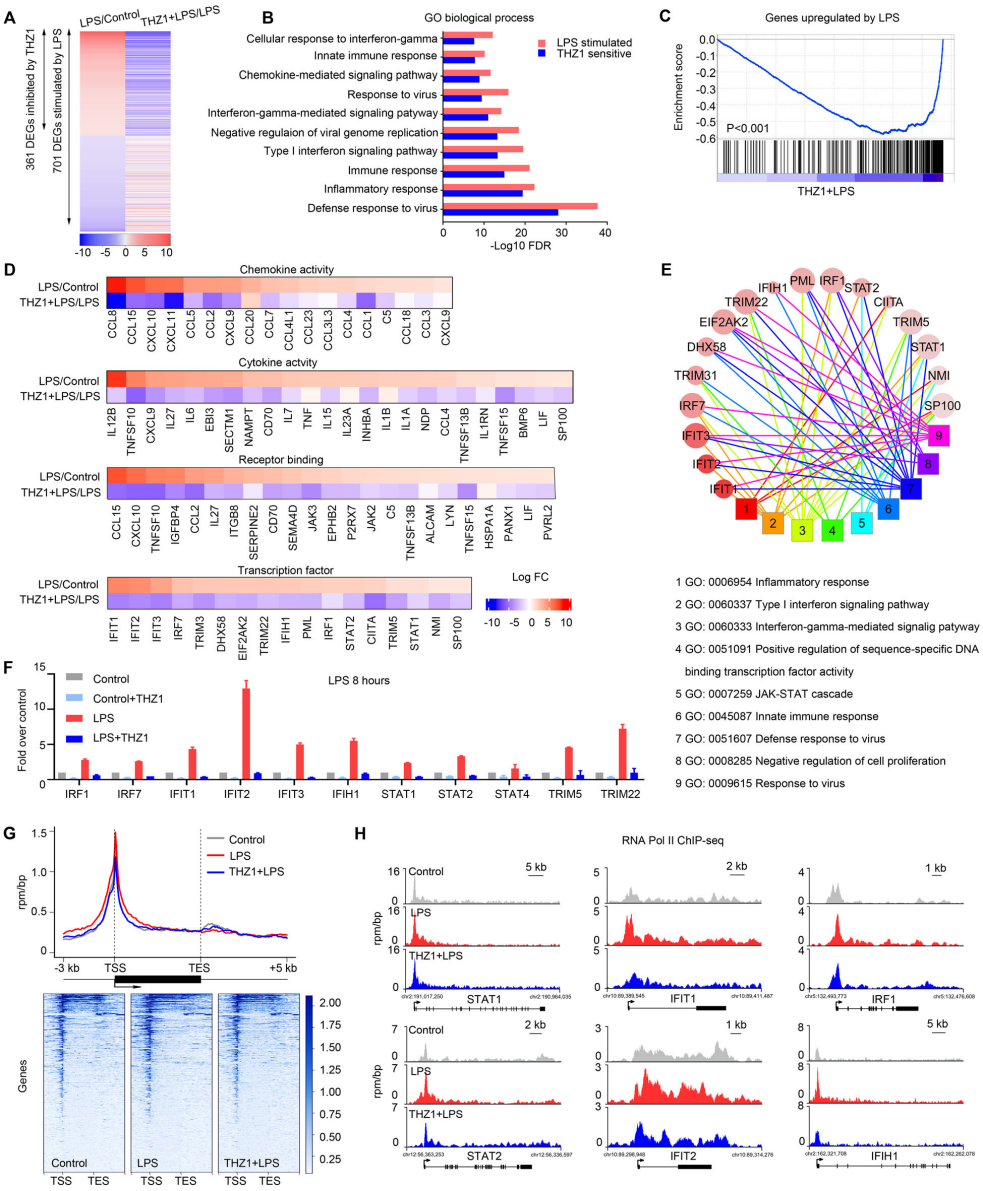
CDK7 inhibitor suppresses the gene profiles of inflammation via RNA Pol II. **a** Heatmap of 701 DEGs in MDMs following 8-h LPS stimulation pretreated with 30 nM THZ1 or not. Heatmap displayed the Log2 fold change in gene expression versus vehicle control or LPS-stimulated cells. **b** The top 10 enriched GO biological processes of 701 LPS-stimulated DEGs and 361 THZ1-sensitive DEGs. Individual bars represent the *P* value after Benjamini-Hochberg correction for enrichment of GO biological processes. **c** There were 361 THZ1-sensitive DEGs enriched using gene set enrichment analysis. **d** Heatmaps of DEGs from the top 3 inhibited GO molecular functions and the TFs described in **e**. **e** Involvement network between upregulated TFs and biological processes. Only the relatively central TFs involved in more than 3 GO terms are shown. **f** Quantitative RT-PCR analysis of partial TFs described in **e**. **g** Peak plot and heatmap of the RNA Pol II ChIP-seq density around the transcription start sites (TSS) and transcription end sites (TES) based on 361 THZ1-sensitive DEGs in control mTHP-1 (gray) and LPS-stimulated mTHP-1 pretreated with THZ1 (blue) or not (red). **h** Gene tracks of RNA Pol II binding density at the representative gene loci after treatment as in **g**. Data are the mean ± SD, *n* = 3 in **f**

The inflammation resolution observed in vitro and in vivo prompted us to further explore the effects of THZ1 on global gene expression. As shown in Fig. 3a, 701 DEGs were significantly changed by LPS stimulation and 361 of the 366 upregulated DEGs could be reversed by THZ1 pretreatment to varying degrees. According to the GO enrichment analysis, we found a high overlap of biological processes between 701 DEGs stimulated by LPS and 361 DEGs that were sensitive to THZ1 (Fig. 3b, Supplementary Table). Pretreatment of THZ1 could precisely reverse the LPS-induced impact on macrophages (Fig. 3c). Importantly, genes encoding chemokines, cytokines and receptors were predominantly suppressed as the top 3 enriched molecules, supporting a relatively broad-spectrum inhibition of THZ1 but limited to inflammatory genes (Fig. 3d). The selective inhibition of THZ1 seemed to achieve a precision strike of inflammatory components. To identify the key regulators in THZ1-mediated repression, we constructed an association network between 52 differentially expressed transcription factors (TFs) and the top 30 enriched GO biological processes according to these THZ1-sensitive DEGs (Supplementary Table). Seventeen TFs were obtained, most of which were well-known in inflammatory regulation, such as STATs, IRFs, IFITs and TRIMs (Fig. 3e). Their transcriptional decline following THZ1 treatment was further validated by quantitative RT-PCR (Fig. 3f). Similar consequences were observed in H1N1-treated macrophages, indicating a common feature of gene profiles triggered by different stimuli (Fig. S3A).

Although THZ1 started to protect against LPS at a low concentration, we performed chromatin immunoprecipitation with high-throughput sequencing (ChIP-seq) to determine whether the decrease in inflammatory transcription correlated with RNA Pol II. We found that THZ1 pretreatment caused a general return to baseline in RNA Pol II binding upstream and in the body of these inflammatory DEGs (Fig. 3g, Fig. S3B). The reduction of RNA Pol II occupancy was also observed in the promoter regions of representative genes, suggesting that their expression was severely curtailed in macrophages (Fig. 3h, Fig. S3C). These findings revealed that THZ1 disrupted the overexpression of inflammatory genes by inhibiting RNA Pol II-mediated transcriptional activity, and the effects might mainly focus on a small group of genes because the dose we used caused no obvious shutdown at the total phosphorylation level of RNA Pol II, which might account for the stability of global transcription.

### CDK7 inhibitor suppresses super enhancer-linked transcription

**Fig. 4:**
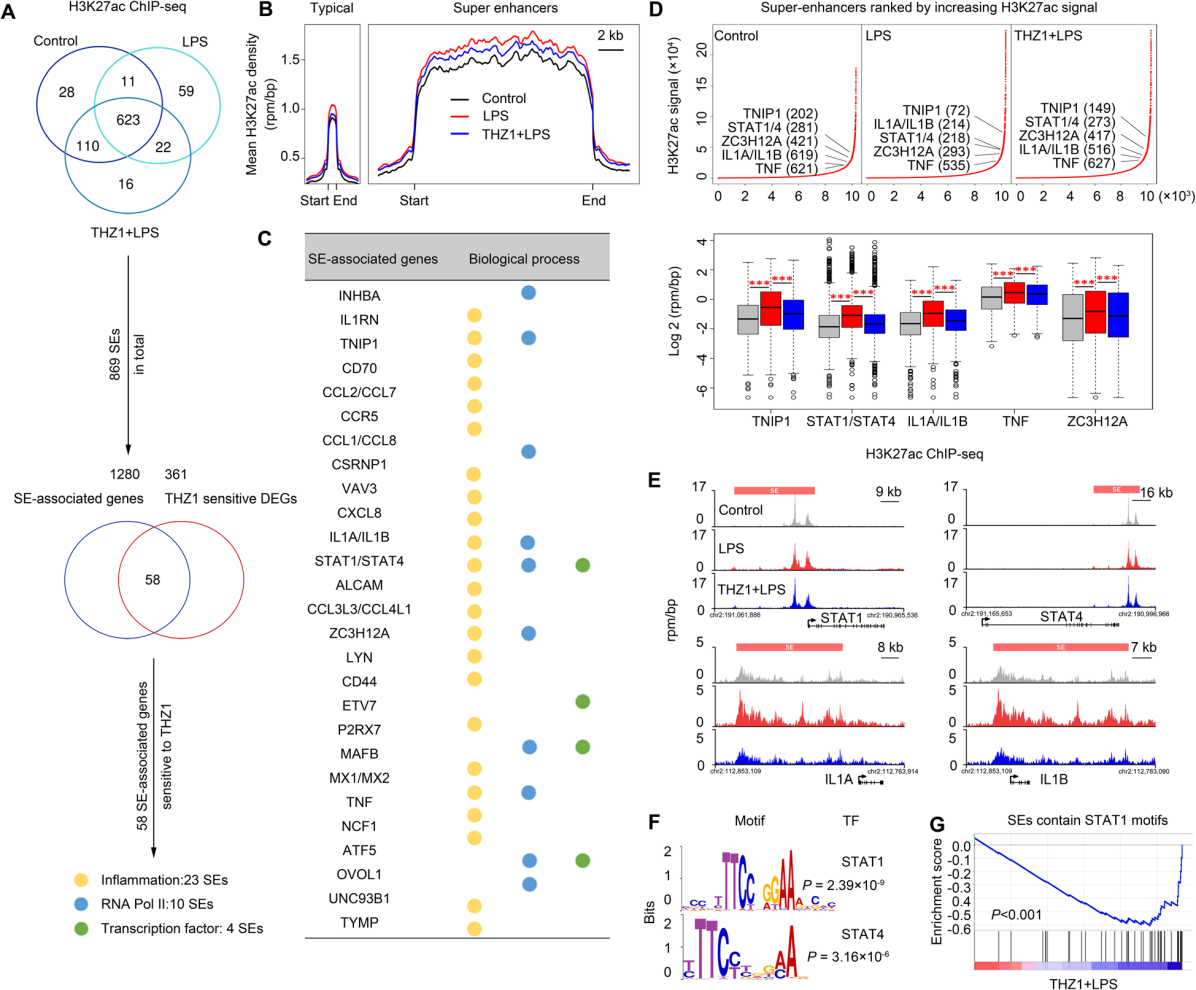
Landscape of super enhancers in macrophages. **a** Venn diagram depicting the overlap between SEs in control mTHP-1 or in LPS-stimulated mTHP-1 pretreated with THZ1 or not. In total, 869 SEs were associated with 1280 genes and 58 genes among them were sensitive to THZ1 pretreatment. **b** Metagene representations of the mean H3K27ac ChIP-seq density for H3K27ac across typical enhancers and SE domains. Metagenes were centered on the enhancer regions, and the length of the enhancer reflected the difference in median lengths of super and typical enhancers. An additional 2 kb surrounding each enhancer region is also shown. **c** Lists of representative SE-associated genes that were sensitive to THZ1. These genes were divided into 3 categories according to their functions. **d** Total H3K27ac signal in enhancer regions for all stitched enhancers in control mTHP-1, LPS-stimulated mTHP-1 pretreated with THZ1 or not. Enhancers were ranked by increasing H3K27ac ChIP-seq signal. SEs of interest are shown along with their respective ranks and their associated genes. **e** ChIP-seq profiles for H3K27ac at the representative SE-associated gene loci in mTHP-1 cells. **f** STAT1 and STAT4 binding motifs enriched at constituent enhancers within SEs and corresponding *P* values. **g** THZ1-sensitive SEs containing STAT1 binding motifs were enriched using gene set enrichment analysis

The transcriptional repression of inflammatory genes raised many questions concerning what determined the genetic susceptibility to THZ1 and the key targets of CDK7-mediated transcription. Accumulating data support that the covalent binding of CDK7 with THZ1 inhibits the phosphorylation of RNA Pol II, which further impacts core transcription factors that are highly marked by acetylation at histone 3 lysine 27 (H3K27ac, a mark of active enhancers), so-called “super enhancers” [29], resulting in a distinctive set of gene regulation that preferentially impedes the high-level expression of cell identity genes [30]. The cooperative properties and synergistic activation of SEs confer disproportionate sensitivity to perturbation so that small changes in SEs lead to dramatic changes in the transcription of related genes [31]. Therefore, we hypothesized a mechanistic explanation for the sensitivity of inflammatory genes to THZ1 and investigated the possible contribution of SEs to the susceptibility of macrophages. Using ChIP-seq of H3K27ac, we identified 869 SEs in total, 772 SEs in control cells and 715 and 771 in activated and rescued cells, respectively (Fig. 4a). The increased H3K27ac level of 869 SEs in activated cells was downregulated with THZ1 pretreatment (Fig. 4b, Fig. S4A). These SEs were associated with 1280 protein-coding genes which were mainly enriched in the inflammatory response (Fig. S4B, Supplementary Table). Among these genes, 58 presented sensitivity to THZ1 pretreatment and were filtered and divided into 3 categories based on their biological processes (Fig. 4a, c, Supplementary Table). We checked the biological importance of these differentially expressed SE-associated genes using the same enrichment analysis above with the 1280 SE-associated genes as background, and we confirmed that THZ1 could primarily affect the SE-associated genes involved in the inflammatory response without interfering the house-keeping gene (Fig. S4B, C). Given the function of CDK7 in phosphorylating RNA Pol II, genes within both categories (inflammation-related functions, regulation of transcription from RNA Pol II promoter) were considered as the key targets of THZ1 treatment, including TNIP1, IL1A/IL1B (regulated by the same SE), STAT1/STAT4 (regulated by the same SE), TNF and ZC3H12A.

Controlled by both SE and RNA Pol II, analysis of ChIP-seq indicated the pivotal transcription factors of STAT1/STAT4 in STATs family (Fig. 4d, e, Fig. S4D), and STAT1 might serve a greater role based on its higher expression (Fig. S4D, E). The binding frequency for known STAT1/STAT4 DNA sequence motifs showed their significant enrichment at SE constituents (Fig. 4f). Despite an absence of total suppression with slight inhibition of CDK7, STAT1 was vulnerable to low-dose THZ1, as well as low-dose SY-1365 and BS-181, especially the dramatic decrease in STAT1 phosphorylation in IFN-γ-treated cells, suggesting the interruption of STAT1-dependent signaling after blocking CDK7 (Fig. S4F). We further analyzed SE-associated genes with STAT1 binding motifs and filtered 43 targets. Enrichment analysis suggested that the transcription of these SEs, which contained STAT1-associated motifs, was also blocked with THZ1 pretreatment (Fig. 4g, Supplementary Table). Additionally, the reduction of H3K27ac signals also confirmed the significant decrease in H3K27ac occupancy for IL1A/IL1B with THZ1 pretreatment (Fig. 4d, e). Our finding revealed a more central role of IL-1 that could be regulated by a specific SE rather than IL-6, the inflammatory function of which has been elucidated in depth [32]. Being upstream of the IL-6 and soluble IL-6 receptor, IL-1 is becoming a more promising target for intervention because IL-1 signaling blockade is speculated to also alleviate the inflammatory response triggered by IL-6 signaling. As we can expect from the central role of STAT1/IL1, pre-inhibition of CDK7 would not only avoid the overexpression of STAT1/IL1 themselves but also continue to suppress associated downstream molecules, resulting in a broad-spectrum inhibition. More importantly, these inhibitory effects mainly focused on inflammatory genes without affecting global transcription. Taken together, the hyperinflammatory state of macrophages is suppressed by regulating the CDK7-dependent transcription addiction of inflammatory genes, leading to a broad-spectrum but selective inhibition.

### THZ1 inhibits CAR T therapy-induced cytokine release

**Fig. 5:**
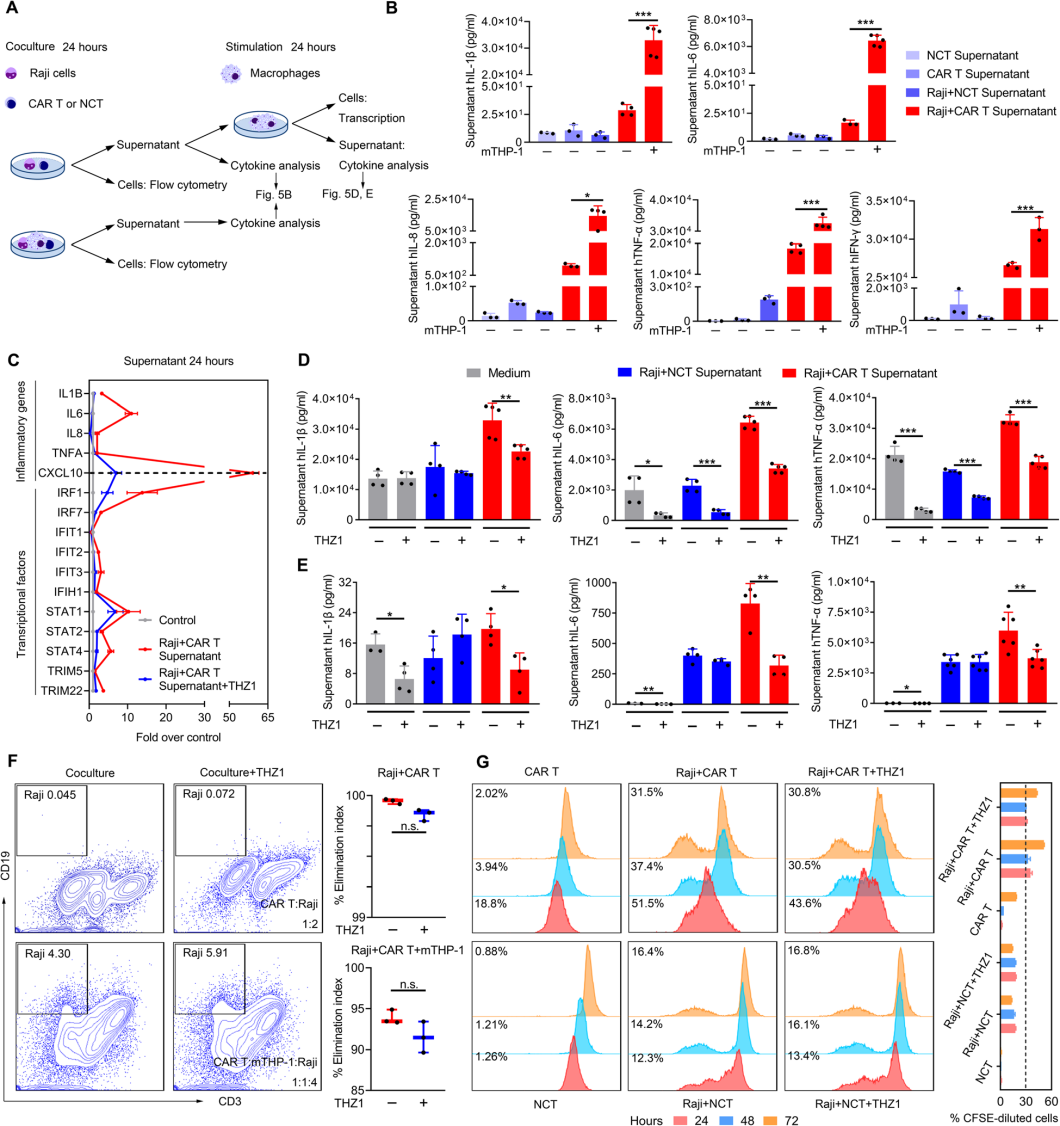
THZ1 inhibits the CAR T cell-induced inflammatory response. **a** Schematic of the experiments in Fig. 5. Coculture of two cell types (Raji + CAR T) and three cell types (Raji + CAR T + macrophages) were performed to obtain the corresponding supernatant and residual cells. The E/T ratio was set as CAR T: Raji = 1: 2 in the coculture of two cell types, CAR T: mTHP-1: Raji = 1: 1: 4 in the coculture of three cell types. 2 × 10^6^ Raji cells were added into these coculture systems to provide equal tumor burden and the total number of all cells remained the same. **b** Human cytokines were detected in the supernatant of NCT cells, CAR T cells, coculture of Raji and NCT cells, coculture of Raji and CAR T cells, and coculture of Raji and CAR T and mTHP-1 cells. **c** Transcriptional levels of inflammatory genes and TFs in mTHP-1 cells cultured with medium, or supernatant of Raji and CAR T cells in the presence of 20 nM THZ1 or not for 24 h. **d** Supernatant levels of human cytokines were detected in mTHP-1 cells cultured with medium, supernatant of Raji and NCT cells, or supernatant of Raji and CAR T cells with or without 20 nM THZ1 for 24 h. **e** Supernatant levels of human cytokines in MDMs. MDMs were treated as in **d**. **f** The residual Raji cells were detected in coculture systems with or without 20 nM THZ1 for 24 h. Representative plots (left) and elimination rates of Raji cells (right) are shown. n.s.: no statistical significance. **g** The E/T ratio was set as CAR T: Raji = 8: 1 in the coculture system. Proliferation of CAR T cells was measured through CFSE dilution after 24, 48 and 72 h. Representative plots (left) and percentages of cells diluted through CFSE (right) are shown. Data are the mean ± SD, n = 3–5 in **b** to **e**. n = 3 in **f** and **g**. ***P < 0.001, **P < 0.01, and *P < 0.05 by the unpaired *t* test in **b**, **d** to **f**

The efficacy and hypotoxicity of THZ1 in mitigating LPS-induced CRS implied a great practical value for clinical application, especially in some cases with a high incidence of CRS such as CAR T cell therapy, which can happen quickly and develop extremely fast in the few days/weeks after infusion. The rapid onset of CRS and short reaction time make prophylactic treatment with THZ1 very maneuverable to prevent the effects of the cytokine storm and minimize the potential hazards following CAR T infusion. Given the approval of 2nd-generation CAR T treatment, human CD19 scFv-4-1BB-CD3ζ CAR T cells were produced with standard quality (Fig. S5A), and cocultured with the Raji cells (expressing CD19) for 24 h to detect the supernatant levels of proinflammatory cytokines (Fig. 5a). Unlike the coculture with normal T cells (noted as negative control T cells, NCT) and Raji cells, we observed a striking increase in IL-1, IL-6, IL-8, TNF-α and IFN-γ in the supernatant of the coculture with CAR T and Raji cells (Fig. 5b). Consistent with a greater contribution of macrophages to CRS development [8, 9], the cytokine levels were much higher when mTHP-1 cells existed in the coculture system (Fig. 5b). We collected supernatant from the coculture of CAR T and Raji cells to activate the macrophages, and then detected the transcription level and cytokine secretion of macrophages (Fig. 5a). Transcriptional amplification stimulated by coculture supernatant was found in inflammatory genes as well as related TFs, whereas these genes were dramatically reduced after exposure to THZ1 (Fig. 5c), consequently leading to reduced cytokine release (Fig. 5d, e). Consistent with the expression changes in inflammatory genes, the excessive phosphorylation of RNA Pol II and STAT1 in activated macrophages was suppressed after blocking CDK7 (Fig. S5B). Pretreatment with THZ1 significantly mitigates the hyperactive inflammatory response to protect against severe CRS.

Except for efficacy, the safety of THZ1 must simultaneously be taken into consideration. No apparent toxicity was found in cells treated with low-dose THZ1, particularly in CAR T cells (Fig. S5C-F). Of note, typical factors derived from CAR T cells, such as TNF-α and IFN-γ which could be adequately triggered when interacting with target cells, were not disturbed at the dose used to inhibit gene expression in macrophages (Fig. S5G, L). The difference in susceptibility indicated a therapeutic window for controlling CRS without impacting CAR T cells. Subsequently, we incubated cells at different effector-to-target (E/T) ratios to calculate the elimination rate according to the percentage of residual Raji cells. Better clearance of target cells was clearly achieved at the higher E/T ratio (Fig. S5H), and the killing capability of effector cells was not weakened with THZ1 pretreatment (Fig. 5f, Fig. S5I). Additionally, macrophages or monocytes were supplemented to enrich the cellular components of coculture system. Consistent with previous data, the dose of pretreatment had little impact on Raji elimination (Fig. 5f, Fig. S5J). CFSE dilution was also performed to examine the proliferative cells, and no significant inhibition was found on NCT or CAR T cell expansion (Fig. 5g, Fig. S5K).

### THZ1 mitigates CAR T-induced CRS without compromising antitumor efficacy

**Fig. 6:**
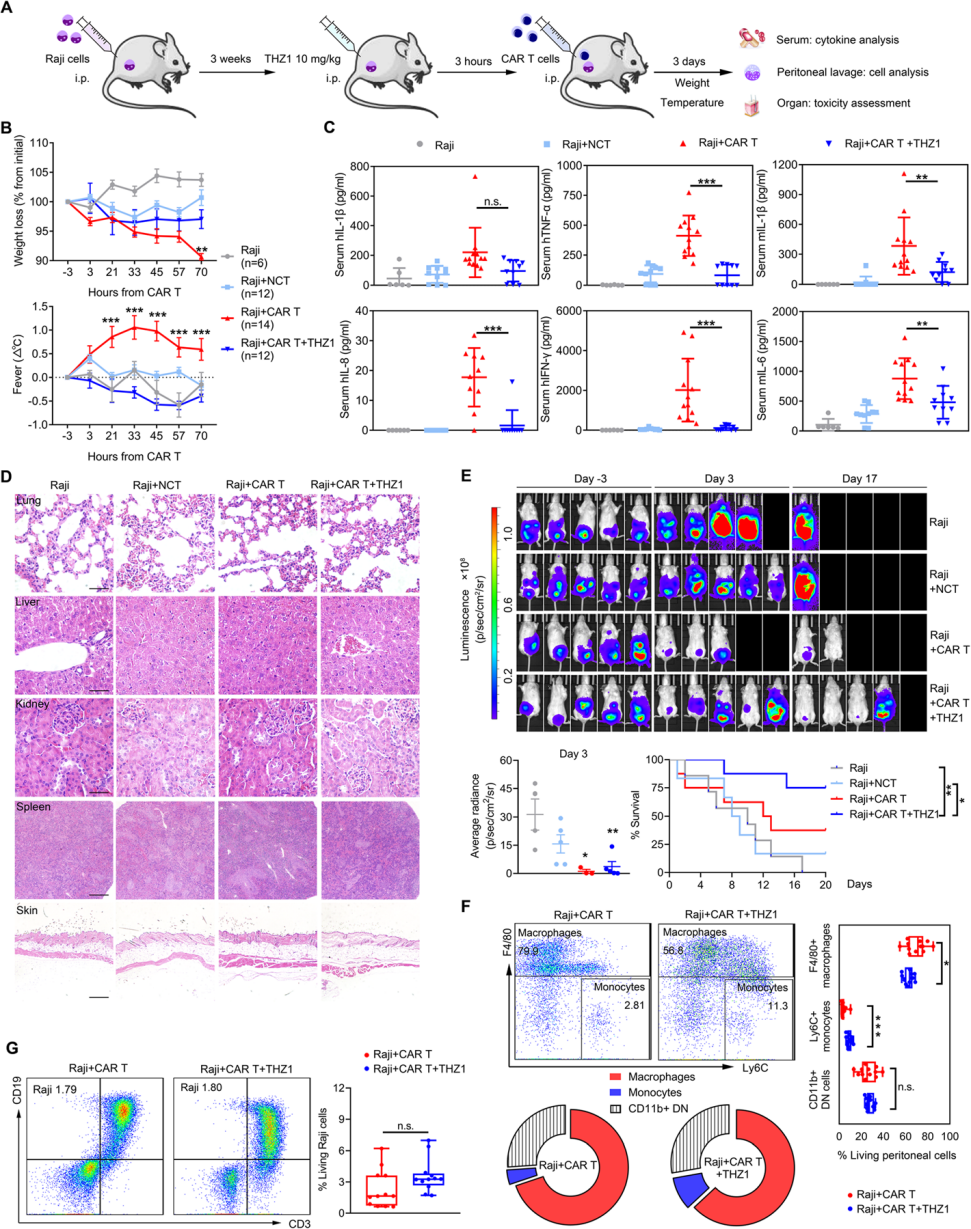
THZ1 mitigates CAR T cell-induced CRS without compromising antitumor efficacy. **a** Schematic of the murine CAR T cell-induced CRS model. Raji cells were intraperitoneally injected into mice and allowed to grow for 3 weeks, after which they were infused following THZ1 pretreatment. **b** Weight and body temperature of tumor-bearing mice after CAR T cell infusion. The weight/temperature of per mouse was normalized to the starting weight/temperature before CAR T infusion. **c** Serum levels of human and murine cytokines from mice at 70 h following CAR T infusion. **d** Tissue sections were obtained from mice on day 3 after CAR T infusion and stained with H&E. **e** Tumor burden and survival of mice after CAR T cell infusion. The one-way ANOVA and the log-rank Mantel-Cox was performed for statistical analysis on day 3 and day 20, respectively. **f** Representative plots and percentages of living F4/80+ macrophages, Ly6C+ monocytes and F4/80-Ly6C- (double negative, DN) myeloid cells in peritoneal lavage. **g** Representative plots and percentages of residual Raji cells in peritoneal lavage. Data are the mean ± SEM, *n* = 6–14 in **b**, *n* = 5-8 in **e**. Data are the mean ± SD, n = 6–14 in **c**, **d** and **f**, **g**. ***P < 0.001, **P < 0.01, and *P < 0.05 by two-way ANOVA in **b**, one-way ANOVA in **c**, **e**, and the unpaired *t* test in **f**, **g**. n.s.: no statistical significance

To assess the therapeutic effects of THZ1 in CAR T-induced CRS, we established a murine model that could develop into severe CRS within 2–3 days according to recent research (Fig. 6a) [9]. Intraperitoneal injection of Raji cells in advance allowed for a sufficient tumor burden to initiate severe CRS in SCID-beige mice. The tolerance of THZ1 pretreatment has been demonstrated at the dose of 10 mg/kg in LPS models and the interval of 3 h in advance was sufficient to pre-inhibit recipient macrophages. After CAR T infusion, the tumor-harboring mice displayed severe weight loss and hyperthermia accompanied by the general presentation of shiver, malaise, and piloerection, but these manifestations could be diminished with THZ1 pretreatment (Fig. 6b). Remarkably, the increased serum levels of cytokines after CAR T infusion, including human IL-1β (hIL-1β), hIL-8, hTNF-α, hIFN-γ, and mIL-1β, mIL-6 [8, 9], could be broadly reduced by THZ1 to alleviate the severity of CRS (Fig. 6c). Histopathological analysis performed 3 days after infusion did not reveal any evidence of graft-versus-host disease (GVHD), which is typically manifested as injury to the skin, mucosa and liver (Fig. 6d). Ki67 and TUNEL staining were also examined in liver and kidney and no overt toxicity was observed with THZ1 treatment (Fig. S6). Despite the powerful antitumor effect of CAR T therapy, the survival benefit was limited due to the acute onset of severe CRS. However, THZ1 pretreatment efficiently mitigated the inflammatory symptoms and improved the survival rate of mice after CAR T infusion (Fig. 6e). Similarly, analysis of peritoneal cells revealed a decrease in CD11b+F4/80+ macrophages after THZ1 pretreatment, concurrent with a slight increase in CD11b+ Ly6C+ monocytes and a steady level of CD11b+F4/80-Ly6C- myeloid cells (Fig. 6f). More importantly, the preventive injection of THZ1 significantly mitigated fatal CRS without impeding the elimination of Raji cells (Fig. 6g). Taken together, these results from in vitro and in vivo CAR T models show the potent suppression achieved with preemptive THZ1 application, indicating THZ1 as an effective candidate for treating CRS.

## Discussion

Severe CRS is an intractable event that requires a novel strategy with better efficacy and safety. After entering the era of immunotherapy, protection against the cytokine storm is indispensable for achieving the therapeutic purpose and controlling the related toxicity. In this study, we assessed the therapeutic potential of the CDK7 inhibitor THZ1 in pathogen-induced CRS models and reconfirmed its protective effects on CAR T cell-induced CRS. Preemptive inhibition of CDK7 significantly suppressed the magnitude of cytokine release and diminished the severity of CRS. Moreover, neither cytotoxicity nor tissue injury was observed in our CRS models with the proper concentrations of THZ1. These findings support the use of THZ1 as a relatively broad-spectrum inhibitor to disrupt excessive inflammatory activity.

Unlike ATP-competitive CDK inhibitors with transient effects, THZ1 leads to a potent and time-dependent transcriptional inhibition because of the prolonged and irreversible CDK7 inactivation caused by the formation of covalent bonds [28]. Due to the potent interruption of functional CDK7, the consequent RNA Pol II dephosphorylation is expected to cause a global shutdown of transcription. Currently, however, an increasing number of studies have improved our understanding of blocking CDK7, which tends to impact a set of genes regulated by specific SEs that determine the genetic or even the cellular response. The CDK7-dependent transcriptional addition reported in MYCN-driven neuroblastoma [15], triple-negative breast cancer [16], and pancreatic cancer [33] is attractive because SE-associated genes are exceptionally inhibited by THZ1 so that targeting CDK7 is conducive to selective targeting. Additionally, some researchers have pointed out that THZ1 can be used to prevent adaptive treatment resistance by inhibiting the drug-induced dynamic transcriptional response and enhancer remodeling, which are required for tumor survival [34, 35]. Despite the different goals of treatments, the susceptibility of SEs to THZ1 indicates a universality of targeting CDK7 to remove the transcription activity induced by SEs, so inhibiting CDK7 might be a potential strategy to significantly reduce the difficulty of managing disease complexity by targeting specific SEs to control aberrant transcription [34, 35]. A similar transcriptional repression was also observed in our study at a much lower dose of THZ1 without disrupting RNA Pol II phosphorylation, that is, global transcription was less disturbed whereas a variety of inflammatory genes were significantly suppressed as a result of the restraint of RNA Pol II phosphorylation in response to stimulation. As expected, the decreased transcription of inflammatory genes was supposed to reduce secondary interactions between different cell types, leading to the resolution of inflammation. In contrast to the dramatic change in macrophages, the slight disturbance of gene transcription revealed a certain tolerance to THZ1 in T and stromal cells, supporting a therapeutic window for targeting CDK7, which seemed to pre-stabilize the vulnerable genes and facilitate reversal of the altered transcription in activated macrophages while having limited effects on other relatively insensitive cells.

**Fig. 7:**
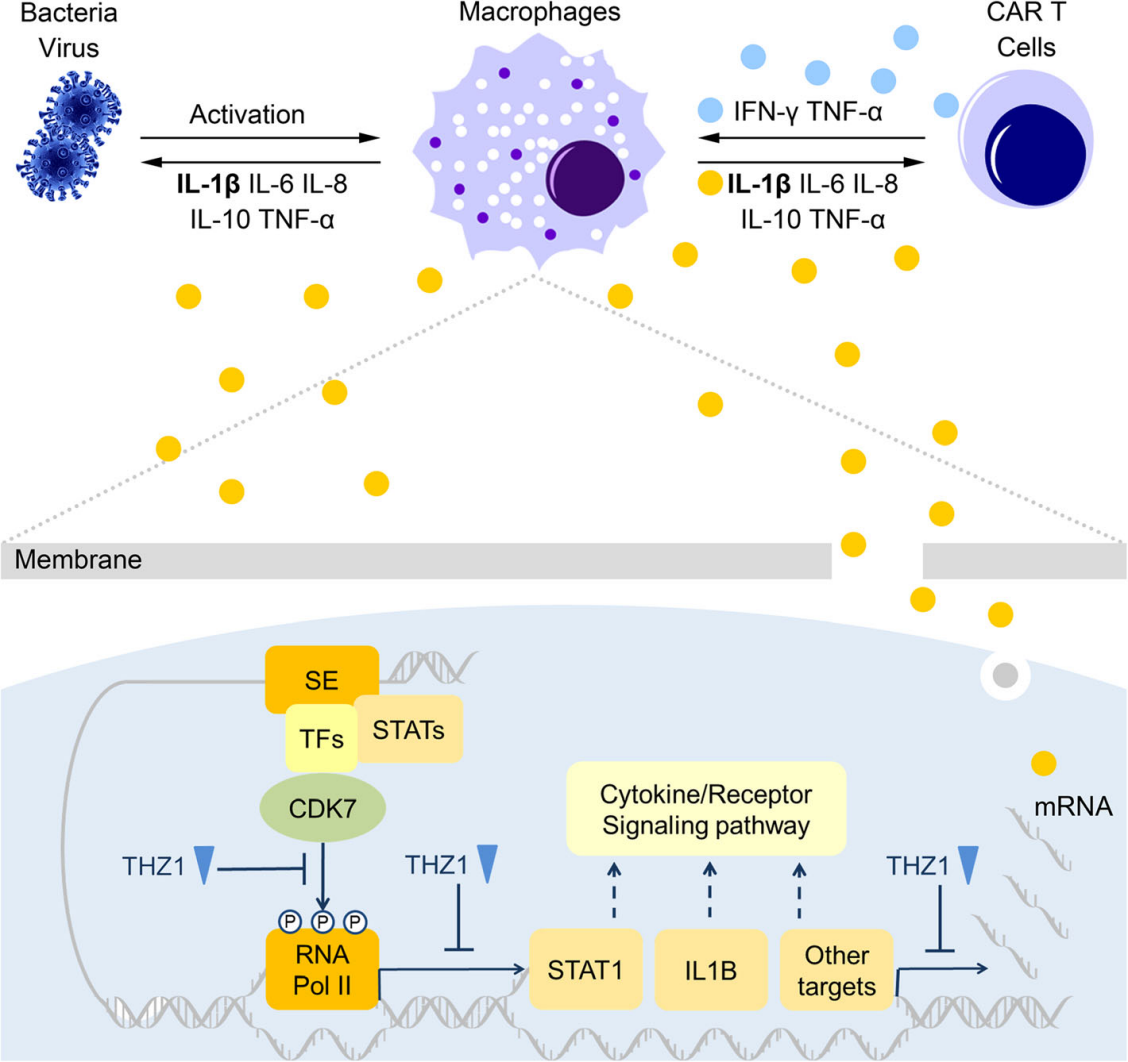
Schematic illustration of the protection of THZ1 during CRS treatment. Overwhelming release of cytokines in activated macrophages contributes to severe CRS. THZ1 can efficiently alleviate the magnitude of cytokine release by binding with CDK7 to reduce the phosphorylation of RNA Pol II after exposure to stimulation, which restricts the massive transcription of inflammatory genes and related transcriptional factors. Among these genes, STAT1 and IL1 are expected to be the key regulator and effector respectively because their transcriptional activities are under the control of super enhancer and influence a variety of crucial downstream molecules such as IL-6. Thus, targeting CDK7 provides a promising candidate for CRS treatment as a relatively broad-spectrum inhibitor

Further insight into the CDK7-associated transcriptional addiction revealed the key targets of THZ1 treatment. Combining RNA- and ChIP-seq data, we showed the genes encoding inflammatory components such as effectors, receptors, or regulators, could be inhibited by CDK7 blockade through RNA Pol II-dependent transcription, and we depicted the landscape of SEs in activated macrophages. Our work examines the priority of STAT1/IL1 in the inflammatory response and suggests that THZ1 blunts the inflammatory cascade by decreased transcription of central STAT1/IL1 and consequent reduction of downstream TFs/genes (Fig. 7). Instead of IL-6, our findings revealed a more essential role of IL-1 and the IL1 receptor antagonist anakinra, which blocks the biological activity of IL-1α and IL-1β by competitively binding to the corresponding receptors and has been approved for attenuating inflammation [36]. Compared with the anti-IL-6 receptor antibody tocilizumab, the current first-line therapy for CAR T-induced CRS fails to prevent CAR T-induced encephalopathy, one advantage of using IL-1 blockade is to reduce systemic concentrations of IL-1 that can cross the blood-brain barrier and cause neurotoxicity [37]. Indeed, several studies have confirmed that IL-1 blockade significantly ameliorates CRS and neurotoxicity without affecting the antileukemic activity of CAR T cells [8, 9]. Great progress has been made in the field of targeting specific inflammatory mediators. Apart from the blockade of IL-1 or IL-6, recent research on lenzilumab, a monoclonal anti-GM-CSF antibody, has shown similar efficacy and safety in moderating CRS-associated injury [38]. Nevertheless, no solo antibody can resolve all the problems associated with CRS given the complexity of the cytokine storm. Broad-spectrum intervention is therefore an optional supplement to fulfill the anti-inflammatory potential of current therapy.

The advent of CAR T has revolutionized the treatment for hematologic malignancies, and thus, more attentions has been focused on enhancing the killing effect of armored CAR T cells by improving the structure and costimulatory domains of CARs. Unfortunately, the killing capacity of CAR T cells is also proportional to the risk of CRS [39, 40]. As expected, increasing challenges will be forthcoming from CRS with the modification of next-generation CAR T [4] and exploration of multitarget immunotherapy [41]. This dilemma will always impede clinical applications if we fail to deal with the cytokine storm. Additionally, the 2019 global outbreak of the novel coronavirus pneumonia (COVID-19) emphasizes the necessity of CRS prevention due to its threat to life [42]. According to recent reports, a similar immunopathologic lesion is described in severe COVID-19 cases, with enriched macrophages in lungs and elevated cytokines such as IFN-γ, IL-1, IL-6, TNF, and IL-18 in blood, accelerating the collapse of immune homeostasis [43]. These discoveries indicate that the strategy used to harness CAR T may be helpful to develop treatments for hyperinflammatory responses in COVID-19 patients. Most prominently, IFN-γ is often more elevated in patients with cytokine storm due to CAR T therapy than in patients with sepsis-induced cytokine storm, who often have higher levels of circulating IL-1β. Serum cytokine levels that are elevated in patients with COVID-19-associated cytokine storm include IL-1β, IL-6, TNF, IFN-γ and MIP-1 [44]. It is plausible to hypothesize that the therapeutic effects of blocking CDK7 would still work in CRS models sharing different cytokine profiles, because the broad-spectrum inhibition of THZ1 makes it applicable for disrupting multiple cytokine-mediated signaling pathways. Of course, adequate confirmations should be implemented carefully to evaluate the feasibility of using THZ1 for more CRS patterns.

There are several limitations of our study. Further explorations are needed to assess the total benefits from specific cytokine blockade or CDK7 inhibition. Additionally, the new highly selective covalent CDK7 inhibitors YKL-5-124 and SY-1365 were recently reported [19, 45], and the comparison between THZ1 and these more selective inhibitors was helpful to identify their mechanistic differences in CDK7-mediated inflammatory regulation. Another challenge for CRS treatment is to identify common points that are shared in various forms of the cytokine storm. Despite the importance of STAT1 and IL1 found in our work, the detailed interactions in inflammatory defense remain to be elucidated.

## Conclusions

We demonstrate that the CDK7 inhibitor THZ1 efficiently ameliorates the inflammatory disorder triggered by LPS, H1N1 and CAR T infusion by suppressing the transcription of inflammatory genes. The potent transcriptional decline of THZ1 is executed via SE-associated inflammatory genes with significant effects on macrophages but negligible effects on other tissues, thus supporting the potential of inhibiting CDK7 for treating CRS.

## Supplementary Information

**Additional file 1.** Supplementary materials and methods.

**Additional file 2: Figure. S1** Supplementary data related to Fig. 1. (**A**) Effects of CDK7 inhibitors on cell proliferation. Cells were treated with the indicated concentrations for the indicated times, and detected using CCK8. (**B**) Effects of CDK7 inhibitors on cell apoptosis. Cells were treated with the indicated concentrations for 48 hours, and detected using Annexin V-FITC/PI flow cytometry. (**C**) Transcriptional levels of inflammatory genes in response to low-dose (100 ng/ml) or high-dose (500 ng/ml) LPS in mTHP-1 cells with 30 nM THZ1 pretreatment or posttreatment at 6 and 24 hours. (**D**) Transcriptional levels of inflammatory genes in response to 500 ng/ml LPS in mTHP-1 cells pretreated with 30 nM THZ1 at 4, 8 and 24 hours. (**E**) Transcriptional levels of inflammatory genes in mTHP-1 cells pretreated with 30 nM SY-1365 or 10 μM BS-181 at 6 hours after LPS stimulation. Data are the mean ± SD, *n* = 3-5 in (A) to (E). ***P < 0.001, **P < 0.01, and *P < 0.05 by one-way ANOVA in (C), unpaired *t* test in (D).

**Additional file 3: Figure S2** Supplementary data related to Fig. 2. (**A**) Survival of mice receiving different doses of LPS. The dose of 40 mg/kg was chosen to induce rapid and severe CRS. (**B**) Tissue sections were obtained from mice after THZ1 pretreatment and stained with H&E. (**C**) The gating strategy to phenotype and FACS sort myeloid populations in cells obtained from peritoneal lavage. Data are the mean ± SD, *n* = 5 in (A) and (B). A log-rank Mantel-Cox was performed for statistical analysis in (A).

**Additional file 4: Figure S3** Supplementary data related to Fig. 3. (**A**) Transcriptional levels of TFs in response to H1N1 infection in mTHP-1 cells pretreated with 30 nM THZ1 at 24 hours. (**B**) Peak plot and heatmap of RNA Pol II ChIP-seq density of 11408 genes in control mTHP-1 and LPS-stimulated mTHP-1 pretreated with THZ1 or not. (**C**) Boxplots of RNA Pol II levels in the ± 1kb around the transcription start sites (TSS) of the inflammatory genes under different conditions. The RNA Pol II signals at most inflammatory genes significantly increased in response to LPS stimulation and decreased with THZ1 pretreatment. ***P < 0.001, **P < 0.01, and *P < 0.05 by the paired *t* test in (C).

**Additional file 5: Figure S4** Supplementary data related to Fig. 4. (**A**) Boxplots of H3K27ac ChIP-seq density for all typical enhancers and SE domains. (**B**) The top 5 enriched GO biological processes of 1280 SE-associated genes or 58 THZ1-sensitive SE-associated genes. (**C**) Boxplots of the H3K27ac signals at 58 THZ1-sensitive SE-associated genes and GAPDH. (**D**) Analysis of the gene expression level, RNA Pol II density, and H3K27ac density at SE regions associated with STAT family. (**E**) H3K27ac density distribution for STAT1-proximal super enhancer in the control, stimulated and rescued cells based on 1000 bins (left). Boxplot for Pol II density at promoter-proximal bins for STAT1 (± 1kb around the annotated start site, upper right). Expression change of STAT1 were presented by RNA-seq and quantitative PCR (low right). (**F**) Western blot analysis of STAT1 and RNA Pol II phosphorylation in mTHP-1 cells treated with 100 ng/ml IFN-γ for 30 minutes following inhibiting CDK7. ***P < 0.001 by the paired *t* test in (A), (C) to (E).

**Additional file 6: Figure S5** Supplementary data related to Fig. 5. (**A**) Schematic of CAR T cell generation. CD25 and CD69 were detected on day 2 to verify the T cell activation. CD3, CD4, and CD8 were examined weekly to monitor the distribution of T subsets. (**B**) Western blot analysis of STAT1 and RNA Pol II phosphorylation in mTHP-1 cells stimulated by the supernatant of coculture with Raji and CAR T cells following 30 nM THZ1 pretreatment for 4 hours. (**C, E**) Effects of THZ1 on cell proliferation. CAR T or NCT cells were treated with indicated concentrations for the indicated times, and detected using the CCK8 kit. (**D, F**) Effects of THZ1 on cell apoptosis. CAR T or NCT cells were treated with indicated concentrations for 48 hours, and detected using flow cytometry. (**G**) Transcriptional levels of inflammatory genes in NCT or CAR T cells treated with 20 nM THZ1 at 24 hours. (**H**) The residual Raji cells were detected in coculture systems with E/T ratio increases from CAR T: Raji = 1: 10 to CAR T: Raji = 1: 2 at 24 hours. (**I**) The residual Raji cells were detected in coculture systems with the E/T ratio NCT: Raji = 1: 2 at 24 hours. Coculture of NCT and Raji cells was set as the control to calculate the elimination rate. (**J**) The residual Raji cells were detected in coculture systems with the E/T ratio CAR T: THP-1: Raji = 1: 1: 4 at 24 hours. (**K**) Proliferation of CAR T or NCT cells in the presence of THZ1 was measured by CFSE dilution after 24, 48 and 72 hours. (**L**) Human cytokines were detected in the supernatant of coculture of Raji and CAR T cells at 24 hours with 20 nM THZ1 or not. n.s.: no statistical significance. Data are the mean ± SD, *n* = 3 in (A) to (L).

**Additional file 7: Figure S6** Supplementary data related to Fig. 6. Sections of liver and spleen were obtained from mice on day 3 after CAR T infusion and stained with Ki67 or with TUNEL. *n* = 3.

**Additional file 8.** Supplementary Table. Lists of genes related to RNA-seq, RNA Pol II ChIP-seq and H3K27ac ChIP-seq.

## Data Availability

All data obtained and/or analyzed during the current study are available from the corresponding authors on reasonable request.

## Abbreviations

CRS: Cytokine release syndrome
CAR T: chimeric antigen receptor engineered T cells
CDK7: Cyclin-dependent kinase 7
IFN: Interferon
IL: Interleukin
CSF: Colony-stimulating factor
TNF: Tumor necrosis factor
SE: Super enhancer
STAT: Signal transducer and activator of transcription
RNA Pol II: RNA polymerase II
GO: Gene Ontology
DEG: Differentially expressed gene
TF: Transcription factor
ChIP: Chromatin immunoprecipitation
H3K27ac: Acetylation at histone 3 lysine 27
PMA: Phorbol 12-myristate 13-acetate
LPS: Lipopolysaccharides
DMEM: Dulbecco’s Modified Eagle Medium
RPMI: Roswell Park Memorial Institute

## References

[1] Shimabukuro-Vornhagen A, Godel P, Subklewe M, Stemmler HJ, Schlosser HA, Schlaak M (2018). Cytokine release syndrome. J Immunother Cancer.

[2] Martinelli G, Boissel N, Chevallier P, Ottmann O, Gökbuget N, Topp MS (2017). Complete hematologic and molecular response in adult patients with relapsed/refractory Philadelphia chromosome–positive B-precursor acute lymphoblastic leukemia following treatment with Blinatumomab: results from a phase II, Single-Arm, Multicenter Study. J Clin Oncol.

[3] Gökbuget N, Dombret H, Bonifacio M, Reichle A, Graux C, Faul C (2018). Blinatumomab for minimal residual disease in adults with B-cell precursor acute lymphoblastic leukemia. Blood.

[4] June CH, O'Connor RS, Kawalekar OU, Ghassemi S, Milone MC (2018). CAR T cell immunotherapy for human cancer. Science.

[5] Schuster SJ, Bishop MR, Tam CS, Waller EK, Borchmann P, McGuirk JP (2019). Tisagenlecleucel in adult relapsed or refractory diffuse large B-cell lymphoma. N Engl J Med.

[6] Hay KA, Hanafi LA, Li D, Gust J, Liles WC, Wurfel MM (2017). Kinetics and biomarkers of severe cytokine release syndrome after CD19 chimeric antigen receptor-modified T-cell therapy. Blood.

[7] Li XL, Liu R, Su X, Pan YS, Han XF, Shao CS, et al. Harnessing tumor-associated macrophages as aids for cancer immunotherapy. Mol Cancer. 2019;18. 10.1186/s12943-019-1102-3 PMC6894344 31805946

[8] Norelli M, Camisa B, Barbiera G, Falcone L, Purevdorj A, Genua M (2018). Monocyte-derived IL-1 and IL-6 are differentially required for cytokine-release syndrome and neurotoxicity due to CAR T cells. Nat Med.

[9] Giavridis T, van der Stegen SJC, Eyquem J, Hamieh M, Piersigilli A, Sadelain M (2018). CAR T cell-induced cytokine release syndrome is mediated by macrophages and abated by IL-1 blockade. Nat Med.

[10] Hay KA (2018). Cytokine release syndrome and neurotoxicity after CD19 chimeric antigen receptor-modified (CAR-) T cell therapy. Br J Haematol.

[11] Davila ML, Riviere I, Wang X, Bartido S, Park J, Curran K (2014). Efficacy and toxicity management of 19-28z CAR T cell therapy in B cell acute lymphoblastic leukemia. Sci Transl Med.

[12] Abbasi M, Mousavi MJ, Jamalzehi S, Alimohammadi R, Bezvan MH, Mohammadi H (2019). Strategies toward rheumatoid arthritis therapy; the old and the new. J Cell Physiol.

[13] Bonifant CL, Jackson HJ, Brentjens RJ, Curran KJ (2016). Toxicity and management in CAR T-cell therapy. Mol Ther Oncolytics.

[14] Nilson KA, Guo J, Turek ME, Brogie JE, Delaney E, Luse DS (2015). THZ1 reveals roles for Cdk7 in co-transcriptional capping and pausing. Mol Cell.

[15] Chipumuro E, Marco E, Christensen CL, Kwiatkowski N, Zhang T, Hatheway CM (2014). CDK7 inhibition suppresses super-enhancer-linked oncogenic transcription in MYCN-driven cancer. Cell..

[16] Wang Y, Zhang T, Kwiatkowski N, Abraham BJ, Lee TI, Xie S (2015). CDK7-dependent transcriptional addiction in triple-negative breast cancer. Cell..

[17] Cayrol F, Praditsuktavorn P, Fernando TM, Kwiatkowski N, Marullo R, Calvo-Vidal MN (2017). THZ1 targeting CDK7 suppresses STAT transcriptional activity and sensitizes T-cell lymphomas to BCL2 inhibitors. Nat Commun.

[18] Christensen CL, Kwiatkowski N, Abraham BJ, Carretero J, Al-Shahrour F, Zhang T (2014). Targeting transcriptional addictions in small cell lung cancer with a covalent CDK7 inhibitor. Cancer Cell.

[19] Zhang H, Christensen CL, Dries R, Oser MG, Deng JH, Diskin B (2020). CDK7 Inhibition Potentiates Genome Instability Triggering Anti-tumor Immunity in Small Cell Lung Cancer. Cancer Cell.

[20] Cartwright JA, Lucas CD, Rossi AG (2019). Inflammation resolution and the induction of granulocyte apoptosis by Cyclin-dependent kinase inhibitor drugs. Front Pharmacol.

[21] Rialdi A, Campisi L, Zhao N, Lagda AC, Pietzsch C, Ho JSY (2016). Topoisomerase 1 inhibition suppresses inflammatory genes and protects from death by inflammation. Science.

[22] Kim D, Paggi JM, Park C, Bennett C, Salzberg SL (2019). Graph-based genome alignment and genotyping with HISAT2 and HISAT-genotype. Nat Biotechnol.

[23] Anders S, Pyl PT, Huber W (2015). HTSeq--a Python framework to work with high-throughput sequencing data. Bioinformatics..

[24] Wang L, Feng Z, Wang X, Zhang X (2010). DEGseq: an R package for identifying differentially expressed genes from RNA-seq data. Bioinformatics..

[25] Benjamini Y, Hochberg Y (1995). Controlling the false discovery rate: a practical and powerful approach to multiple testing. J R Stat Soc Ser B Methodol.

[26] Whyte WA, Orlando DA, Hnisz D, Abraham BJ, Lin CY, Kagey MH (2013). Master transcription factors and mediator establish super-enhancers at key cell identity genes. Cell.

[27] Gust J, Hay KA, Hanafi LA, Li D, Myerson D, Gonzalez-Cuyar LF (2017). Endothelial activation and blood-brain barrier disruption in neurotoxicity after adoptive immunotherapy with CD19 CAR-T cells. Cancer Discov.

[28] Kwiatkowski N, Zhang T, Rahl PB, Abraham BJ, Reddy J, Ficarro SB (2014). Targeting transcription regulation in cancer with a covalent CDK7 inhibitor. Nature..

[29] Hnisz D, Abraham BJ, Lee TI, Lau A, Saint-Andre V, Sigova AA (2013). Super-enhancers in the control of cell identity and disease. Cell..

[30] Hah N, Benner C, Chong LW, Yu RT, Downes M, Evans RM (2015). Inflammation-sensitive super enhancers form domains of coordinately regulated enhancer RNAs. Proc Natl Acad Sci U S A.

[31] Hnisz D, Shrinivas K, Young RA, Chakraborty AK, Sharp PA (2017). A phase separation model for transcriptional control. Cell..

[32] Kang S, Tanaka T, Narazaki M, Kishimoto T (2019). Targeting Interleukin-6 signaling in clinic. Immunity..

[33] Lu P, Geng J, Zhang L, Wang Y, Niu N, Fang Y (2019). THZ1 reveals CDK7-dependent transcriptional addictions in pancreatic cancer. Oncogene..

[34] Carugo A, Draetta GF (2018). Collapsing the tumor ecosystem: preventing adaptive response to treatment by inhibiting transcription. Cancer Discov.

[35] Rusan M, Li K, Li Y, Christensen CL, Abraham BJ, Kwiatkowski N (2018). Suppression of adaptive responses to targeted Cancer therapy by transcriptional repression. Cancer Discov.

[36] Nikfar S, Saiyarsarai P, Tigabu BM, Abdollahi M (2018). Efficacy and safety of interleukin-1 antagonists in rheumatoid arthritis: a systematic review and meta-analysis. Rheumatol Int.

[37] Santomasso BD, Park JH, Salloum D, Riviere I, Flynn J, Mead E (2018). Clinical and biological correlates of neurotoxicity associated with CAR T-cell therapy in patients with B-cell acute lymphoblastic leukemia. Cancer Discov.

[38] Sterner RM, Sakemura R, Cox MJ, Yang N, Khadka RH, Forsman CL (2019). GM-CSF inhibition reduces cytokine release syndrome and neuroinflammation but enhances CAR-T cell function in xenografts. Blood..

[39] Raje N, Berdeja J, Lin Y, Siegel D, Jagannath S, Madduri D (2019). Anti-BCMA CAR T-cell therapy bb2121 in relapsed or refractory multiple myeloma. N Engl J Med.

[40] Lindner SE, Johnson SM, Brown CE, Wang LD (2020). Chimeric antigen receptor signaling: Functional consequences and design implications. Sci Adv.

[41] MacKay M, Afshinnekoo E, Rub J, Hassan C, Khunte M, Baskaran N (2020). The therapeutic landscape for cells engineered with chimeric antigen receptors. Nat Biotechnol.

[42] Huang C, Wang Y, Li X, Ren L, Zhao J, Hu Y (2020). Clinical features of patients infected with 2019 novel coronavirus in Wuhan, China. Lancet.

[43] Giamarellos-Bourboulis EJ, Netea MG, Rovina N, Akinosoglou K, Antoniadou A, Antonakos N (2020). Complex immune Dysregulation in COVID-19 patients with severe respiratory failure. Cell Host Microbe.

[44] Fajgenbaum DC, Longo DL, June CH (2020). Cytokine Storm. N Engl J Med.

[45] Hu S, Marineau JJ, Rajagopal N, Hamman KB, Choi YJ, Schmidt DR (2019). Discovery and characterization of SY-1365, a selective, covalent inhibitor of CDK7. Cancer Res.

