# Supplementary material for: Targeting CDK7 suppresses super enhancer-linked inflammatory genes and alleviates CAR T cell-induced cytokine release syndrome
Source: Mol Cancer. 2021 Jan 4;20:5. doi: 10.1186/s12943-020-01301-7 (PMC7780220; doi:10.1186/s12943-020-01301-7)
Supplement: Supplementary file 1 — Additional file 1. Supplementary materials and methods. [file 12943_2020_1301_MOESM1_ESM.docx]

Targeting CDK7 suppresses super enhancer-linked inflammatory genes

and alleviates CAR T cell-induced cytokine release syndrome

**Supplementary materials and methods**

**Drugs and antibodies**

THZ1 (APE×BIO) was dissolved in a 1:9:10 ratio of DMSO, PEG 300 and ddH2O at a storage concentration of 10 mM. LPS (Sigma) was dissolved in 0.9% NaCl at a storage concentration of 5 mg/ml for experiments, cells were stimulated with LPS at the concentration of 500 ng/ml *in vitro* unless otherwise stated. PMA (Sigma) was dissolved in DMSO at a storage concentration of 1mg/ml. All reagents were stored at -20 ℃ and diluted into indicated concentrations before use.

Anti-RNA Pol II (Abcam, 1:1000), anti- RNA Pol II p-Ser 2 (Active motif, 1:1000), anti- RNA Pol II p-Ser 7 (Millipore, 1:1000), anti-STAT1 p-Tyr701 (CTS, 1:1000), anti-STAT1 (CST, 1:1000), anti-CDK7 (Proteintech, 1:2000), anti-β actin (Proteintech, 1:4000) were used according to the manufacture’s instructions.

**Primers**

Sequences of primers used for quantitative RT-PCR were as follows:

IL1B forward: 5’-ATGATGGCTTATTACAGTGGCAA-3’

IL1B reverse: 5’-GTCGGAGATTCGTAGCTGGA-3’

IL6 forward: 5’-ACTCACCTCTTCAGAACGAATTG-3’

IL6 reverse: 5’-CCATCTTTGGAAGGTTCAGGTTG-3’

IL8 forward: 5’-TTTTGCCAAGGAGTGCTAAAGA-3’

IL8 reverse: 5’-AACCCTCTGCACCCAGTTTTC-3’

TNFA forward: 5’-GGAGAAGGGTGACCGACTCA-3’

TNFA reverse: 5’-TGCCCAGACTCGGCAAAG-3’

CXCL10 forward: 5’-GTGGCATTCAAGGAGTACCTC-3’

CXCL10 reverse: 5’-TGATGGCCTTCGATTCTGGATT-3’

IRF1 forward: 5’-ATGCCCATCACTCGGATGC-3’

IRF1 reverse: 5’-CCCTGCTTTGTATCGGCCTG-3’

IRF7 forward: 5’-GCTGGACGTGACCATCATGTA-3’

IRF7 reverse: 5’-GGGCCGTATAGGAACGTGC-3’

IFIT1 forward: 5’-TTCGGAGAAAGGCATTAGA-3’

IFIT1 reverse: 5’-TCCAGGGCTTCATTCATAT-3’

IFIT2 forward: 5’-AGGCTTTGCATGTCTTGG-3’

IFIT2 reverse: 5’-GAGTCTTCATCTGCTTGTTGC-3’

IFIT3 forward: 5’-TCAGAAGTCTAGTCACTTGGGG-3’

IFIT3 reverse: 5’-ACACCTTCGCCCTTTCATTTC-3’

IFIH1 forward: 5’-TCACAAGTTGATGGTCCTCAAGT-3’

IFIH1 reverse: 5’-CTGATGAGTTATTCTCCATGCCC-3’

STAT1 forward: 5’-CAGCTTGACTCAAAATTCCTGGA-3’

STAT1 reverse: 5’-TGAAGATTACGCTTGCTTTTCCT-3’

STAT2 forward: 5’-CCAGCTTTACTCGCACAGC-3’

STAT2 reverse: 5’-AGCCTTGGAATCATCACTCCC-3’

STAT4 forward: 5’-TGTTGGCCCAATGGATTGAAA-3’

STAT4 reverse: 5’-GGAAACACGACCTAACTGTTCAT-3’

TRIM5 forward: 5’-TGAGCAACTGAGAGACATCCT-3’

TRIM5 reverse: 5’-CCGTCCTTTTTATGACGCCATC-3’

TRIM22 forward: 5’-AATGTGCTGGATAACCTGGCA-3’

TRIM22 reverse: 5’-TCTACTGACGATCCCCTCAAC-3’

IFNG forward: 5’-TCGGTAACTGACTTGAATGTCCA-3’

IFNG reverse: 5’-TCGCTTCCCTGTTTTAGCTGC-3’

β-actin forward: 5’-ACCTTCTACAATGAGCTGCG-3’

β-actin reverse: 5’-CCTGGATAGCAACGTACATGG-3’

**Western blot analysis**

Lysates were prepared from cells and quantified using BCA assay kit. Samples were electrophoresed through 8-12 % SDS-PAGE gel and transferred to PVDF membranes. The blots were probed with appropriate primary antibodies. After incubation with secondary antibodies, membranes were washed and stained with ECL according to the manufacturer’s protocol.

**H&E staining and IHC**

Paraffin-embedded sections of tissues were dewaxed in xylene and dehydrated through alcohols, followed by H&E staining to judge tissue toxicity. Ki67 or TUNEL staining was performed to detect the liver and spleen toxicity.
