# Supplementary figures and images for: Targeting CDK7 suppresses super enhancer-linked inflammatory genes and alleviates CAR T cell-induced cytokine release syndrome
Source: Mol Cancer. 2021 Jan 4;20:5. doi: 10.1186/s12943-020-01301-7 (PMC7780220; doi:10.1186/s12943-020-01301-7)

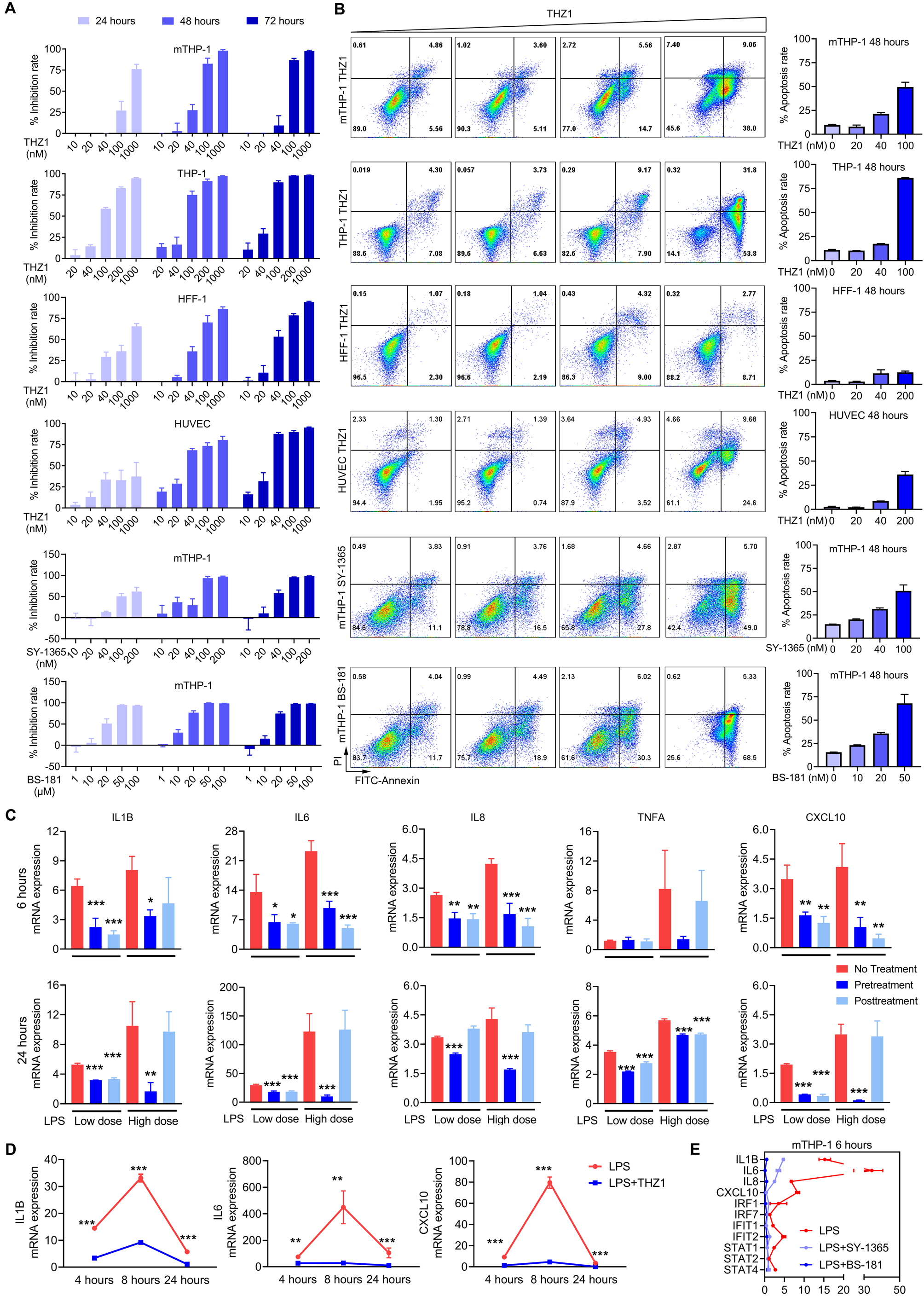

Supplement: Supplementary file 2 — Additional file 2: Figure. S1 Supplementary data related to Fig. 1. (A) Effects of CDK7 inhibitors on cell proliferation. Cells were treated with the indicated concentrations for the indicated times, and detected using CCK8. (B) Effects of CDK7 inhibitors on cell apoptosis. Cells were treated with the indicated concentrations for 48 hours, and detected using Annexin V-FITC/PI flow cytometry. (C) Transcriptional levels of inflammatory genes in response to low-dose (100 ng/ml) or high-dose (500 ng/ml) LPS in mTHP-1 cells with 30 nM THZ1 pretreatment or posttreatment at 6 and 24 hours. (D) Transcriptional levels of inflammatory genes in response to 500 ng/ml LPS in mTHP-1 cells pretreated with 30 nM THZ1 at 4, 8 and 24 hours. (E) Transcriptional levels of inflammatory genes in mTHP-1 cells pretreated with 30 nM SY-1365 or 10 μM BS-181 at 6 hours after LPS stimulation. Data are the mean ± SD, n = 3-5 in (A) to (E). ***P < 0.001, **P < 0.01, and *P < 0.05 by one-way ANOVA in (C), unpaired t test in (D). [file 12943_2020_1301_MOESM2_ESM.tif]

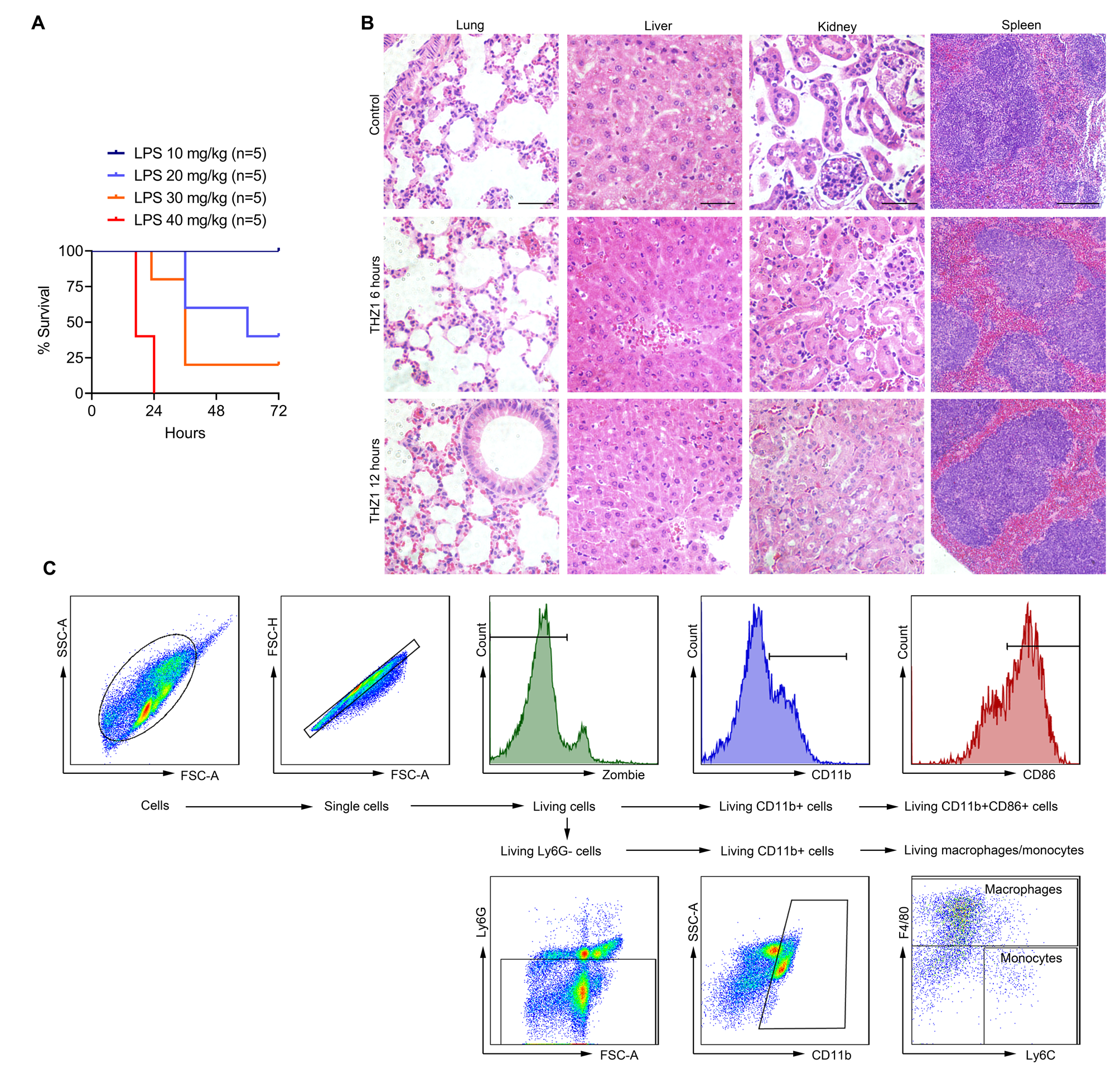

Supplement: Supplementary file 3 — Additional file 3: Figure S2 Supplementary data related to Fig. 2. (A) Survival of mice receiving different doses of LPS. The dose of 40 mg/kg was chosen to induce rapid and severe CRS. (B) Tissue sections were obtained from mice after THZ1 pretreatment and stained with H&E. (C) The gating strategy to phenotype and FACS sort myeloid populations in cells obtained from peritoneal lavage. Data are the mean ± SD, n = 5 in (A) and (B). A log-rank Mantel-Cox was performed for statistical analysis in (A). [file 12943_2020_1301_MOESM3_ESM.tif]

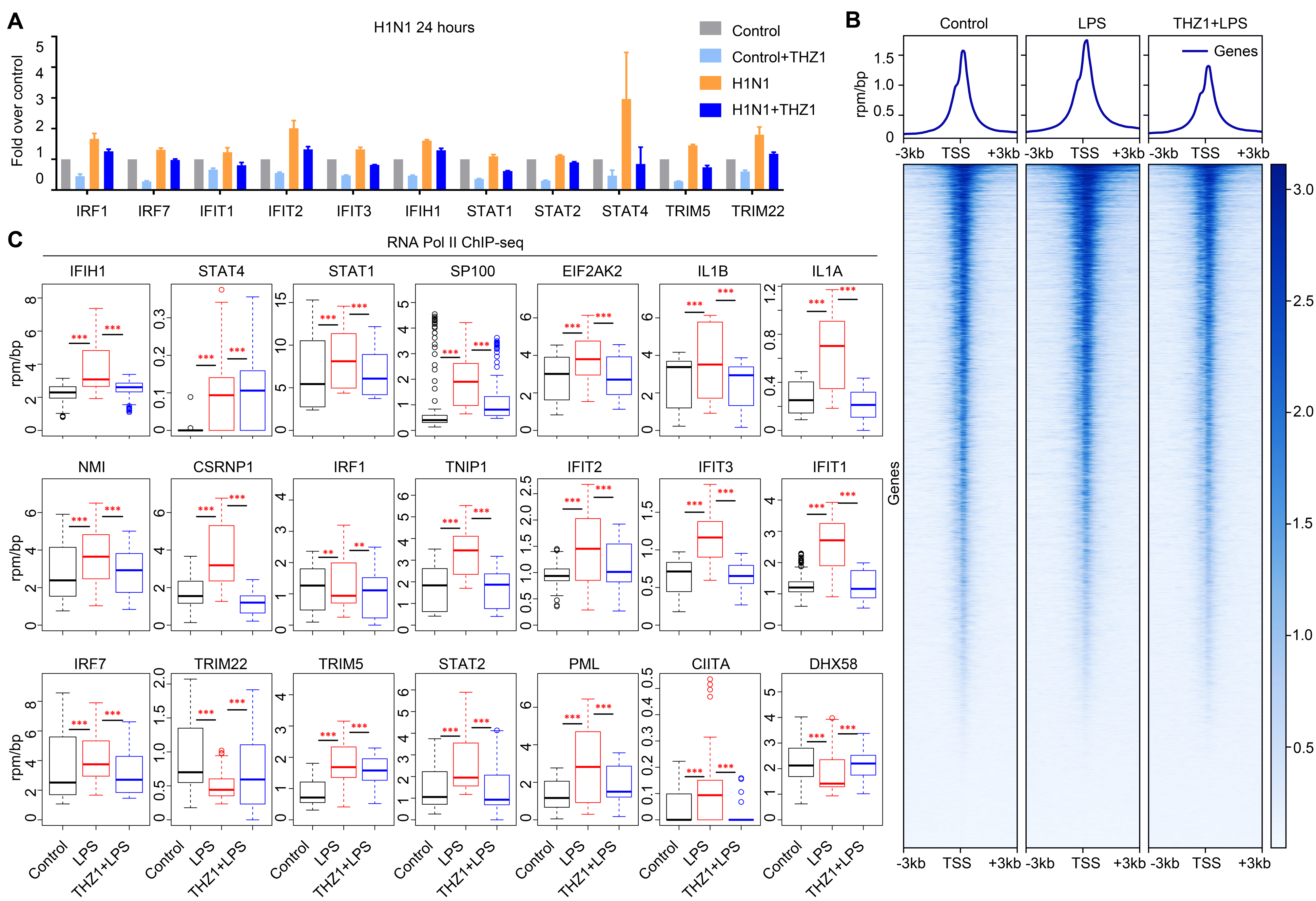

Supplement: Supplementary file 4 — Additional file 4: Figure S3 Supplementary data related to Fig. 3. (A) Transcriptional levels of TFs in response to H1N1 infection in mTHP-1 cells pretreated with 30 nM THZ1 at 24 hours. (B) Peak plot and heatmap of RNA Pol II ChIP-seq density of 11408 genes in control mTHP-1 and LPS-stimulated mTHP-1 pretreated with THZ1 or not. (C) Boxplots of RNA Pol II levels in the ± 1kb around the transcription start sites (TSS) of the inflammatory genes under different conditions. The RNA Pol II signals at most inflammatory genes significantly increased in response to LPS stimulation and decreased with THZ1 pretreatment. ***P < 0.001, **P < 0.01, and *P < 0.05 by the paired t test in (C). [file 12943_2020_1301_MOESM4_ESM.tif]

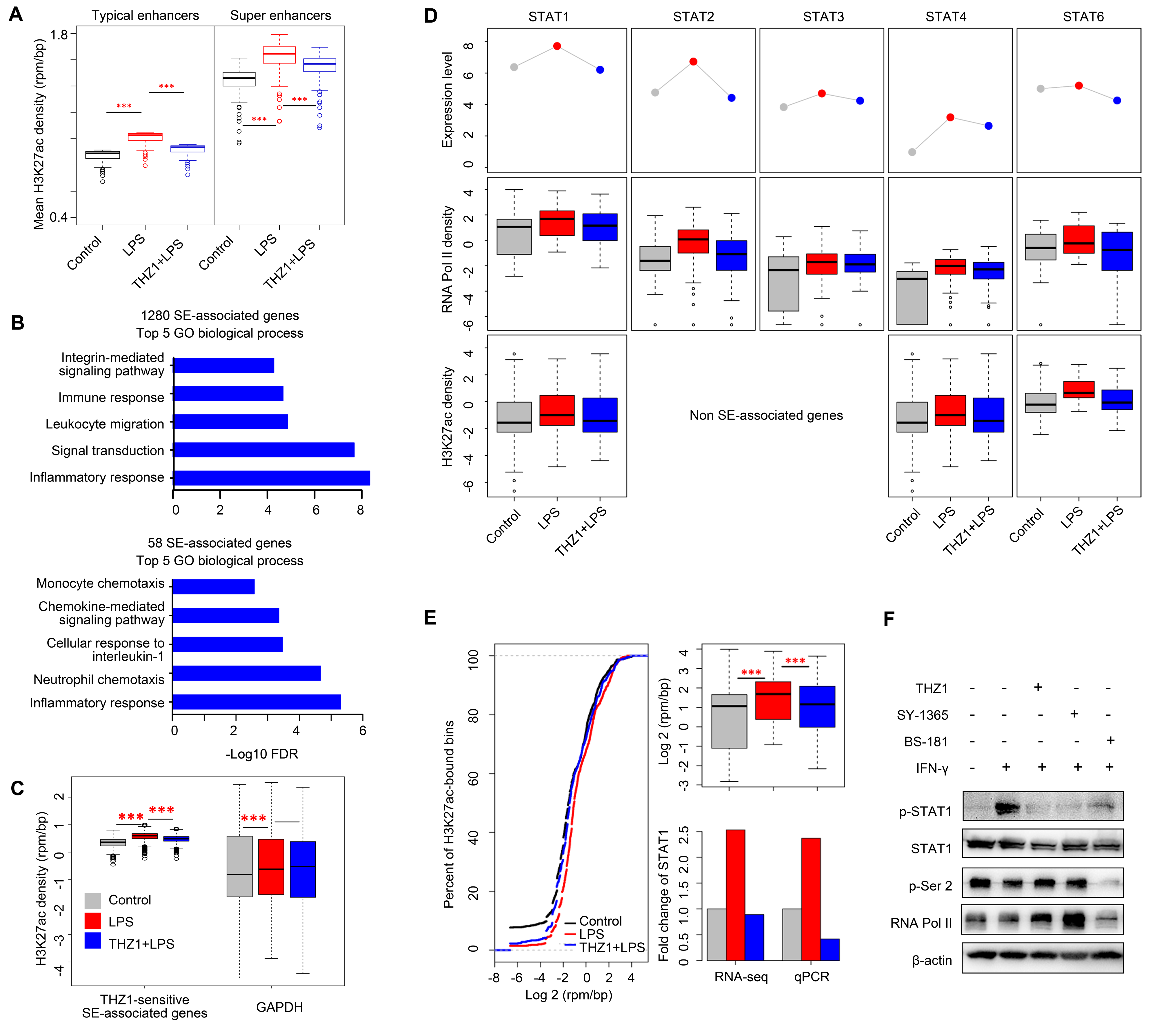

Supplement: Supplementary file 5 — Additional file 5: Figure S4 Supplementary data related to Fig. 4. (A) Boxplots of H3K27ac ChIP-seq density for all typical enhancers and SE domains. (B) The top 5 enriched GO biological processes of 1280 SE-associated genes or 58 THZ1-sensitive SE-associated genes. (C) Boxplots of the H3K27ac signals at 58 THZ1-sensitive SE-associated genes and GAPDH. (D) Analysis of the gene expression level, RNA Pol II density, and H3K27ac density at SE regions associated with STAT family. (E) H3K27ac density distribution for STAT1-proximal super enhancer in the control, stimulated and rescued cells based on 1000 bins (left). Boxplot for Pol II density at promoter-proximal bins for STAT1 (± 1kb around the annotated start site, upper right). Expression change of STAT1 were presented by RNA-seq and quantitative PCR (low right). (F) Western blot analysis of STAT1 and RNA Pol II phosphorylation in mTHP-1 cells treated with 100 ng/ml IFN-γ for 30 minutes following inhibiting CDK7. ***P < 0.001 by the paired t test in (A), (C) to (E). [file 12943_2020_1301_MOESM5_ESM.tif]

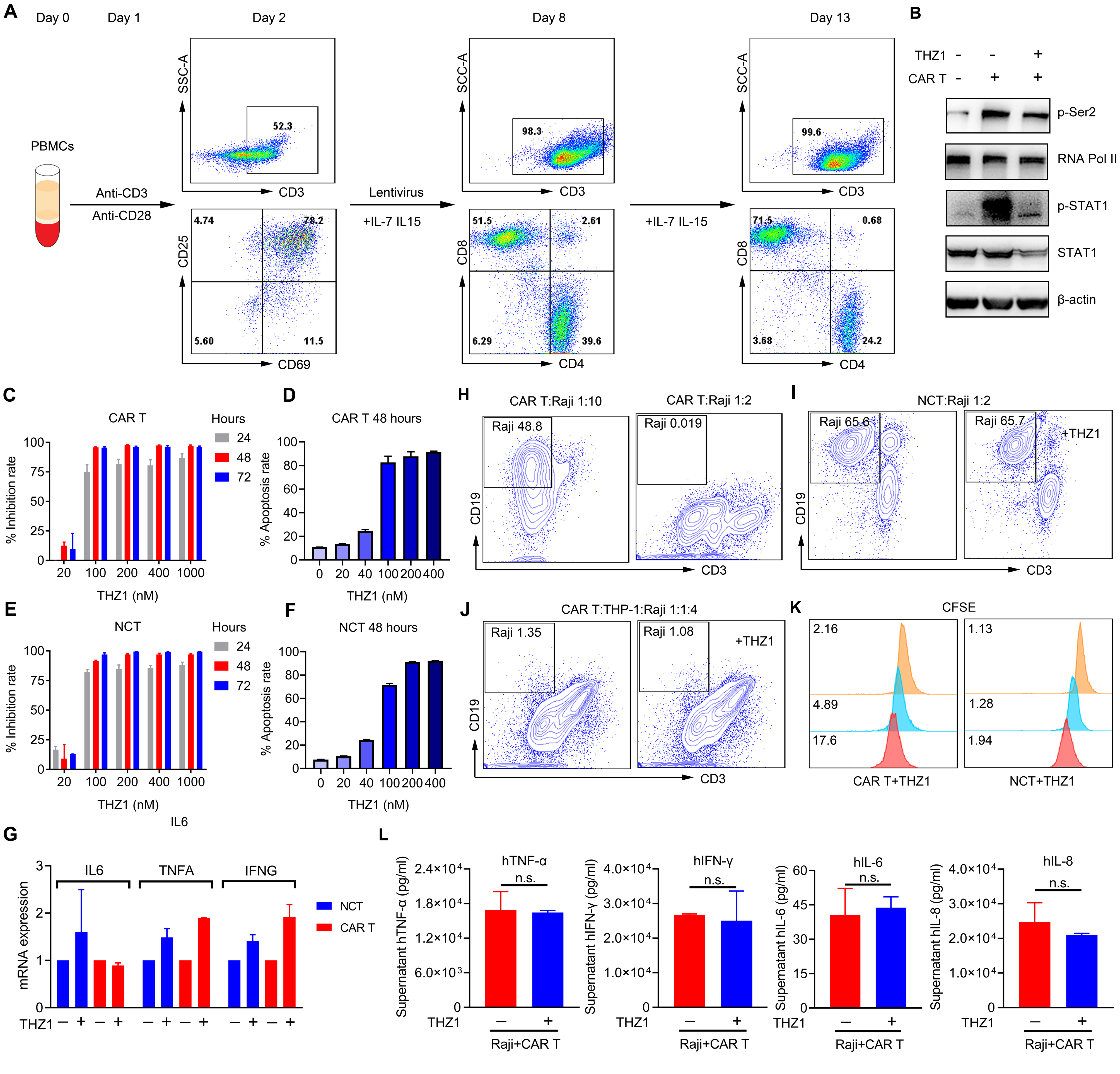

Supplement: Supplementary file 6 — Additional file 6: Figure S5 Supplementary data related to Fig. 5. (A) Schematic of CAR T cell generation. CD25 and CD69 were detected on day 2 to verify the T cell activation. CD3, CD4, and CD8 were examined weekly to monitor the distribution of T subsets. (B) Western blot analysis of STAT1 and RNA Pol II phosphorylation in mTHP-1 cells stimulated by the supernatant of coculture with Raji and CAR T cells following 30 nM THZ1 pretreatment for 4 hours. (C, E) Effects of THZ1 on cell proliferation. CAR T or NCT cells were treated with indicated concentrations for the indicated times, and detected using the CCK8 kit. (D, F) Effects of THZ1 on cell apoptosis. CAR T or NCT cells were treated with indicated concentrations for 48 hours, and detected using flow cytometry. (G) Transcriptional levels of inflammatory genes in NCT or CAR T cells treated with 20 nM THZ1 at 24 hours. (H) The residual Raji cells were detected in coculture systems with E/T ratio increases from CAR T: Raji = 1: 10 to CAR T: Raji = 1: 2 at 24 hours. (I) The residual Raji cells were detected in coculture systems with the E/T ratio NCT: Raji = 1: 2 at 24 hours. Coculture of NCT and Raji cells was set as the control to calculate the elimination rate. (J) The residual Raji cells were detected in coculture systems with the E/T ratio CAR T: THP-1: Raji = 1: 1: 4 at 24 hours. (K) Proliferation of CAR T or NCT cells in the presence of THZ1 was measured by CFSE dilution after 24, 48 and 72 hours. (L) Human cytokines were detected in the supernatant of coculture of Raji and CAR T cells at 24 hours with 20 nM THZ1 or not. n.s.: no statistical significance. Data are the mean ± SD, n = 3 in (A) to (L). [file 12943_2020_1301_MOESM6_ESM.tif]

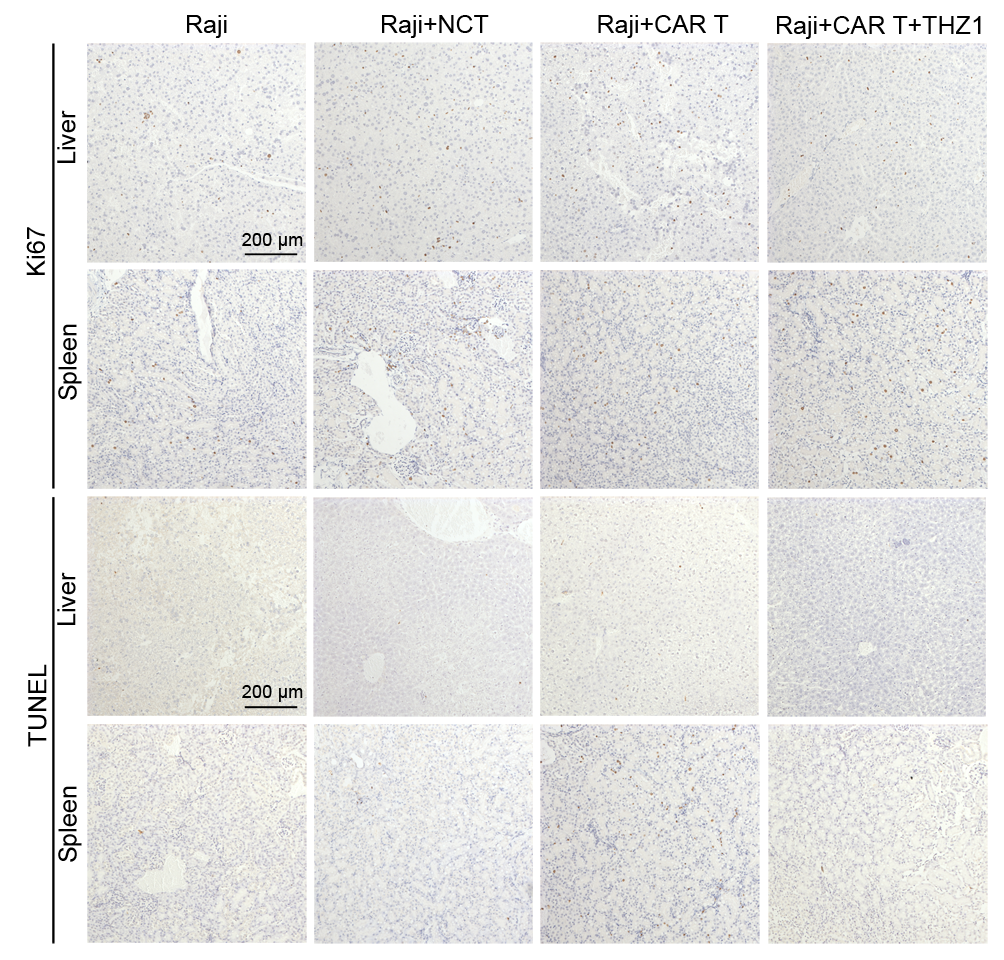

Supplement: Supplementary file 7 — Additional file 7: Figure S6 Supplementary data related to Fig. 6. Sections of liver and spleen were obtained from mice on day 3 after CAR T infusion and stained with Ki67 or with TUNEL. n = 3. [file 12943_2020_1301_MOESM7_ESM.tif]
